# A Preliminary Evaluation of Advanced Oxidation Protein Products (AOPPs) as a Potential Approach to Evaluating Prognosis in Early-Stage Breast Cancer Patients and Its Implication in Tumour Angiogenesis: A 7-Year Single-Centre Study

**DOI:** 10.3390/cancers16051068

**Published:** 2024-03-06

**Authors:** Marta Napiórkowska-Mastalerz, Tomasz Wybranowski, Maciej Bosek, Stefan Kruszewski, Piotr Rhone, Barbara Ruszkowska-Ciastek

**Affiliations:** 1 Department of Biophysics, Faculty of Pharmacy, Nicolaus Copernicus University, Collegium Medicum, 85-067 Bydgoszcz, Poland; tomaszwybranowski@cm.umk.pl (T.W.); mbosek@cm.umk.pl (M.B.); skrusz@cm.umk.pl (S.K.); 2 Clinical Ward of Breast Cancer and Reconstructive Surgery, Oncology Centre Prof. F. Łukaszczyk Memorial Hospital, 85-796 Bydgoszcz, Poland; rhonep@co.bydgoszcz.pl; 3 Department of Pathophysiology, Faculty of Pharmacy, Nicolaus Copernicus University, Collegium Medicum, 85-094 Bydgoszcz, Poland

**Keywords:** early-stage breast cancer, AOPP, adjuvant treatment, overall survival, disease-free survival

## Abstract

**Simple Summary:**

Breast cancer is the main cause of cancer-related deaths among women globally. It clearly shows it is essential to find a useful marker, which could have a beneficial influence on the prediction of cancer-related events and give more information about the possible outcomes in the early stage of the disease. This is the first study that presents advanced oxidation protein products as a potentially valuable biomarker for monitoring the course of breast cancer treatment and as a predictor of recurrence and cancer-related mortality. The study shows also the relationship between oxidative protein damage in breast cancer patients and excessive angiogenic processes.

**Abstract:**

Breast cancer (BrC) is a highly prevalent tumour among women. The high incidence and mortality rate of BrC prompts researchers to search for new markers that will provide information on the possible impact of the therapy on the risk of cancer-related events. This study aimed to investigate whether the level of advanced oxidation protein products (AOPPs) may have a potential impact on disease-free (DFS) and overall survival (OS) in BrC patients with early-stage cancer. Additionally, we tried to assess the relationship between AOPPs and angiogenic parameters. In this study, the pre- and post-treatment AOPP levels were examined in the serum of 70 newly diagnosed BrC women. The receiver operating characteristic curve identified pre- and post-treatment AOPPs to be above 9.37 μM and 10.39 μM, respectively, as the best cut-off values to predict the risk of cancer relapse. Additionally, Kaplan–Meier survival analysis indicated that pre- and post-treatment AOPPs above 9.37 μM and 10.39 μM were associated with significantly poorer OS. The uni- and multivariate Cox regression analysis highlighted that lower levels of pre- and post-treatment AOPPs were associated with a longer duration without relapse or cancer-related death. A positive correlation between concentrations of pre-treatment AOPPs and vascular endothelial growth factor A, and negative correlations with levels of soluble forms of vascular endothelial growth factor receptor type 1 and 2, were found. In conclusion, AOPPs appear to have an important role in predicting cancer-related events and may potentially serve as a simple prognostic marker in clinical practice.

## 1. Introduction

Breast cancer (BrC) is considered the most commonly diagnosed cancer worldwide, representing 11.7% of total cases, and the leading cause of cancer-associated death among women. The global report shows that in 2020 there were an estimated almost 2.3 million new cases of female breast cancer, with about 685,000 cancer-related deaths [1,2]. The 5-year relative survival from diagnosis is 90.6%; however, this is strongly associated with the cancer stage at diagnosis [3]. The risk of progression in the early stage of breast cancer is estimated to be 20–30% [4]. To discover a biomarker that could be useful in predicting such events, there is a need to understand the underlying mechanisms of tumour development. Breast cancer cells, like typical tissue, need a sufficient supply of oxygen and constant nourishment for growth. Hypoxia induced by a growing tumour triggers various types of responses, mediated among others by hypoxia-inducible factors (HIFs). Subsequently, HIFs regulate the expression of numerous types of genes, which are responsible for stimulating specific physiological processes, including metabolism, angiogenesis, and cell division [5]. 

Angiogenesis is a process which leads to the creation of new blood vessels from pre-existing ones. Under physiological conditions, angiogenesis plays a crucial role in wound healing and normal growth. However, tumour growth and metastasis are also strongly dependent on angiogenesis. The interplay between pro- and anti-angiogenic factors is of considerable importance in the tight regulation of angiogenesis. It is believed that some specific tumour cells produce both pro- and anti-angiogenic proteins that stimulate and inhibit angiogenesis, respectively. Some researchers have assumed that tumour cells alter the balance between pro- and anti-angiogenic factors, which leads to the activation of the angiogenic switch [5,6,7]. Continuous pro-angiogenic stimulation in the tumour environment leads to the formation of immature, poorly organized, and malformed vessels, which express enhanced permeability. All these processes are related to unequal blood flow and persisting hypoxia, and may induce the progression of the tumour [8]. There are found several pro-angiogenic factors engaged in stimulating vascular growth. The main regulator of angiogenesis and tumour growth is regarded as the vascular endothelial growth factor (VEGF) family, inclusive of seven isoforms VEGF-A/B/C/D/E and placental growth factor (PIGF) 1 and 2. In blood, there are three forms of soluble receptors secreted by endothelial cells (sVEGFR1, sVEGFR2, sVEGFR3) [9]. A soluble form of VEGFR1 binds to VEGF-A, which expresses the highest biological activity among the VEGF family in breast cancer patients. A soluble form of VEGFR1 naturally inhibits endothelial cell proliferation induced by VEGF [9,10]. VEGF also forms non-signalling ligands with sVEGFR2, thus slowing their migration and proliferation and, therefore, the formation of new blood vessels in malignant tumours [11,12]. 

One of the most essential factors influencing the intensity of the angiogenic processes is oxidative stress, defined as the imbalance between the production of reactive oxygen species (ROS) and antioxidant defences. Excessive ROS production plays a key role in the vascularization of cancerous tissue and metastasis. Increased ROS production with ageing, smoking, obesity, and alcohol consumption, which are possible risk factors for developing breast cancer, also supporting their involvement in this process [13]. Chronic inflammation induced by oxidative stress leads to oxidative damage to DNA, proteins, and lipids, which in turn contributes to the dysregulation of oncogenes and tumour suppressor genes, e.g., p53, BRCA2, and finally to the initiation and progression of the tumour [7,14]. The level of oxidative stress can alternatively be examined by the amount of damage acted on proteins by measuring the concentration of advanced oxidation protein products (AOPPs). AOPPs are usually formed from albumins and methionine- or tyrosine-rich polypeptides as a consequence of ROS attacks [15]. The elevated levels of AOPPs found by some researchers were related to ageing, diabetes, atherosclerosis, and metabolic syndrome [16,17,18,19]. Moreover, the involvement of oxidative stress on proteins has been also found in COVID-19 pneumonia patients [20]. Zhou et al. have also suggested that an increased concentration of AOPPs may serve as a predictor of mortality in haemodialysis patients [21]. There are some investigations describing associations between AOPPs and angiogenic biomarkers. One study revealed the relationship between elevated levels of AOPPs and higher expressions and secretions of VEGF and sVEGFR1 in trophoblast cell lines. This finding shows the interplay between increased AOPP concentrations and endothelial and placental angiogenesis dysfunction [19]. Liu et al. have also indicated a strong link between the stimulation of AOPPs and triggering of the proliferation and migration of rat endometrial epithelial cells along with restraining apoptosis by activation of the ERK and P38 pathways [22]. Furthermore, Le-Wu et al. have revealed that elevated levels of AOPPs in colon cancer cells were closely associated with an increased expression of VEGF exposed to intermittent hypoxia [23]. Additionally, AOPPs may also directly stimulate angiogenesis by promoting oxidative stress and inflammation [24,25]. A study by Guo et al. even suggests that AOPPs can activate vascular endothelial cells through a specific signalling pathway mediated by a receptor for advanced glycation end products (RAGE) [26]. 

It has been demonstrated that oxidative stress can enhance angiogenesis [7,27]. However, mechanistic insights into the underlying molecular mechanisms that connect AOPPs and VEGF have not been yet fully presented. Several hypotheses can be proposed to elucidate the potential interactions of these two markers. The development of tumours is a complex process in which angiogenesis plays a crucial role, enabling tumours to grow and metastasize through the formation of new blood vessels. As the tumour progresses, tumour cells are subjected to various forms of stress, including hypoxia, which can lead to their death through necrosis or apoptosis, consequently releasing DNA into the extracellular environment, known as circulating cell-free DNA (cfDNA) [28,29,30,31,32]. In breast cancer, it has also been shown that an increase in circulating tumour DNA (ctDNA) (the fraction of cfDNA that originates from tumour cells) can be a good indicator and may be used to monitor disease progression and response to treatment in patients with advanced breast cancer [33]. However, beyond its role as a diagnostic and prognostic marker, cfDNA can also contribute to pathological inflammation and disease progression, acting through various immunological and molecular mechanisms. Circulating tumour DNA can be adeptly recognized by the immune system as a damage-associated molecular pattern (DAMP) [34,35,36,37,38]. This recognition triggers a series of immune responses, including the activation of neutrophils and the release of neutrophil extracellular traps (NETs) [39,40,41]. A significant enzyme in this context is myeloperoxidase (MPO), a heme-containing enzyme abundantly found in the azurophilic granules of neutrophils [42]. MPO is crucial for the oxidative burst, a process generating ROS [43,44]. This oxidative stress is intimately linked to the formation of AOPP, primarily through the action of hypochlorous acid (HOCl), produced by MPO [45,46]. Our in vitro studies using human serum albumin (HSA) demonstrated that chloramine T, a source of active chlorine substituting unstable HOCl, induces the formation of AOPPs-HSA in a dose-dependent exponential manner [20].

The integration of AOPPs in the angiogenesis process and their interaction with RAGE adds another layer of complexity to the already complicated picture of tumour development and progression [24,47]. The binding of AOPPs to RAGE receptors can lead to the activation of transcription factors such as NF-κB, which regulate the expression of genes involved in the inflammatory response and initiate signalling cascades leading to the expression and secretion of various cytokines, including IL-6 and TNFα. Such an increase in inflammatory activity can promote further tumour development, creating an environment conducive to angiogenesis. In the same study, the blockade of RAGE signals significantly suppressed aggressive behaviour and inflammatory response. Additionally, on cell lines, it was shown that AOPPs additionally induce the expression of matrix metalloproteinase-3 (MMP-3) and MMP-13 [25]. MMPs are involved in the degradation of extracellular matrix components, which not only facilitates the migration and invasion of tumour cells but can also modulate the release and activation of growth factors associated with the extracellular matrix. MMPs can participate in releasing matrix-bound forms of VEGF, increasing its bioavailability and promoting angiogenesis. By degrading extracellular matrix components, MMPs can facilitate the release of VEGF deposited in the matrix, enabling its interaction with receptors on endothelial cells, and stimulating angiogenesis [48]. This evidence firmly establishes a direct correlation between VEGF and AOPPs, suggesting that the angiogenic processes driven by VEGF are closely linked to oxidative stress and inflammation mediated by AOPPs. Such a connection underscores the complexity of tumour microenvironment interactions and highlights potential therapeutic targets for disrupting the pathological feedback loops that fuel cancer progression. However, the role of AOPPs in cancer-related angiogenesis is still not well understood, and further research is needed to clarify this relationship.

This is the first study showing the impact of AOPP concentration in the serum of early-stage breast cancer patients on disease-free and overall survival before and after treatment. Thus, the aim of this study was to examine the usefulness of AOPPs as a prognostic marker of the likely course of the disease in a group of women with breast cancer, using ROC curve analysis, Kaplan–Meier curves, and Cox regression analysis. Additionally, we sought to assess the influence of the adjuvant treatment on the blood serum concentration of AOPPs in breast cancer patients and to demonstrate a relationship between AOPPs and selected angiogenic biomarkers. Furthermore, we explored associations between AOPP levels and clinical and molecular characteristics of BrC. 

## 2. Materials and Methods

### 2.1. Patient Samples and Clinical Data

**Figure 1:**
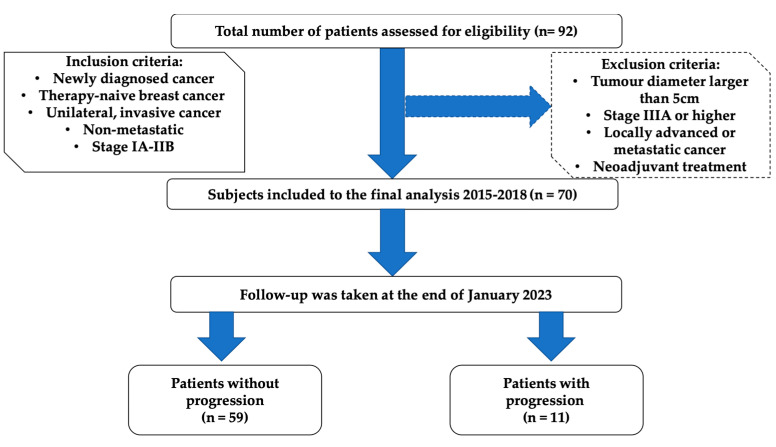
Flowchart describing enrolment of breast cancer participants.

This study was conducted according to the guidelines of the Declaration of Helsinki and approved by the Ethics Committee of Nicolaus Copernicus University in Torun, Collegium Medicum in Bydgoszcz (approval no. KB/547/2015). All included patients gave written informed consent before their enrolment. The study group consisted of 70 previously non-treated women with newly diagnosed breast cancer (BrC). Subjects were enrolled between November 2015 and January 2018 by an oncologist and treated in the Clinical Ward of Breast Cancer and Reconstructive Surgery, Oncology Centre in Bydgoszcz, Poland. Inclusion and exclusion criteria are presented in Figure 1. 

The traditional clinical and histological factors and molecular determinants were examined in all the women included in the study. The participants underwent breast-conserving surgery (BCS) (58 cases; 82.9%) or mastectomy (12 cases; 17.1%), respectively, and received adjuvant chemotherapy, radiotherapy, brachytherapy, immunotherapy, and endocrine therapy if needed. The patient’s TNM stage of cancer was characterised according to the American Joint Committee on Cancer (AJCC; 7th edition). The WHO classification was used to establish histological subtypes of cancer, whereas the Elston–Ellis system was used to classify histological grade. All participants were subjected to thorough evaluation in the form of complete demographic and medical history, and collected data are displayed in Table 1.

### 2.2. Adjuvant Therapy

Adjuvant treatment was administered according to standard guidelines established by the National Comprehensive Cancer Network (NCCN) Guidelines for Practice. Radiotherapy was applied mainly for patients who underwent BCS within 1–2 weeks after the adjuvant chemotherapy completion. The study group received a median dose of 42.5 gray (Gy) in 17–20 fractions over 4–6 weeks to the chest wall by using X photons with energies of 6/15 MeV. Using a direct electron field, a sequential boost of 10/12.5 Gy was administered to the initial tumour bed in 17 patients (24.3%) in four/five fractions. In thirty-six subjects (51.4%), additional brachytherapy in a dose of 10 Gy was applied. Thirty-five subjects (50.0%) received adjuvant chemotherapy. Twenty-five (35.7%) patients had anthracycline-containing drugs administered and non-anthracycline-containing drugs were used in ten patients (14.3%). In fifty-eight subjects (82.9%), endocrine therapy was applied according to menopausal status; 35 (50.0%) used tamoxifen (Egis Pharmaceuticals, Budapest, Hungary), 13 of patients (18.6%) received aromatase inhibitors (AIs) (Arimidex (anastrozole), AstraZeneca, Cambridge, United Kingdom), and in 8 patients (11.4%) the combination of tamoxifen and AIs was given, but 2 patients (2.9%) underwent another endocrine scheme, in an adjuvant setting. In twelve subjects (17.1%), endocrine therapy was not administered due to a small tumour diameter or a triple-negative (ER−/PR−/HER2−/Ki67−all values) subtype of BrC.

### 2.3. Patients Follow-Up

The patients were followed up from the day of the diagnosis of BrC until the date of recurrence of breast cancer or death or until January 2023, whichever came first. Kaplan–Meier curves were created to present overall and disease-free survival. The median follow-up was 78 months (IQR = 70–84 months), with a 15.7% recurrence rate. The follow-up visits after nine months from surgical procedures were performed for each patient.

### 2.4. Blood Collection

The blood collection occurred two times, 24 h before the surgical procedure (pre-treatment value) and a maximum of three months after the last cytotoxic infusion and generally nine months (interquartile range, IQR = 6.0–10.0) after tumour resection (post-treatment value) to avoid the direct effects of chemotherapy or surgical wound healing on the analysed parameters. The blood from each patient was collected into serum-separating tubes to determine the AOPP concentration. After collection, the samples were mixed and centrifuged at 3000× *g* at 4 °C for 15 min, divided into small tubes, and stored at −80 °C until assayed. The blood was also drawn into EDTA-containing tubes for measurements of VEGF-A and soluble forms of VEGF receptor types 1 and 2. The blood samples were immediately mixed and centrifuged at 3000× *g* at 4 °C for 20 min. The collected plasma was aliquoted in small portions and stored at −80 °C until each measurement.

### 2.5. Measurements of AOPP

The AOPP level in serum was determined, according to the modified method reported by Witko-Sarsat, by absorbance measurements at 340 nm [49]. The AOPP detection was modified, in agreement with Hanasand’s improved method, by using citric acid instead of acetic acid, which improves the stability of the sample over time [50]. The first step of the procedure was preparing a reactant mixture containing 1.875 mL of 0.2 M citric acid and 25 µL of 1.16 M potassium iodide. Afterwards, the mixture in the volume of 1.9 mL was mixed with 100 µL of serum. The absorbance was measured immediately after preparation. The results were expressed in µM as chloramine T equivalents. 

### 2.6. Measurements of Angiogenic Factors

The concentrations of angiogenic parameters were determined according to the procedure described in the previous study [51]. The subjects were divided into groups with low or high values of angiogenic biomarkers by using a cut-off based on the calculated median for the study group.

### 2.7. Statistical Analysis

Statistical analysis was conducted using Statistica version 13.3 (StatStoft^®^, Cracow, Poland). The normality of the distribution was checked by using the Shapiro–Wilk test. The unpaired Student’s *t*-test was used to compare two groups of continuous data with normal distribution. The univariate ANOVA analysis, followed by a post hoc LSD (least significant difference) test, was applied to compare more than two groups of continuous data. Comparisons of means for two dependent variables were performed by paired Student’s *t*-test. AOPP data are presented as mean and standard deviation. Spearman’s rank correlation analysis was used to determine dependencies between investigated parameters. The receiver operating characteristic (ROC) was used to utilise the area under a curve (AUC) to estimate the best predictor of cancer relapse. Youden’s method was applied to define the optimal cut-off values. The disease-free survival (DFS) and overall survival (OS) analysis were expressed using the Kaplan–Meier method and compared using the log-rank test. DFS and OS were calculated as times from randomization until breast cancer recurrence or death, respectively. The uni- and multivariate Cox proportional hazard analysis was conducted to estimate cohort-specific hazard ratios (HRs) and 95% confidence intervals (CIs) for the association between AOPP levels and other clinical variables with disease-free and overall survival time to identify a possible predictor that has a significant influence on these times. AOPP concentration was also included in multiple linear regression models with adjustments for BMI, age, parity, menopausal status, smoking status, tumour stage, tumour diameter, histological type, and nodal involvement. Additionally, to emphasize the strength of AOPPs as a prognostic marker of disease recurrence (Figure S1) and cancer-related death (Figure S2), we compared the diagnostic accuracy of AOPPs with Ki-67 and the tumour diameter using the ROC curves. The statistical data were considered significant with *p* < 0.05.

## 3. Results

### 3.1. Effect of Undergone Procedures, Clinical, and Molecular Characteristics on AOPP Concentration

The studied group, enrolled between November 2015 and June 2017, consisted of 70 women with resected breast cancer (BrC) in the early stage (IA-IIB). The median age of subjects at the diagnosis was 54 years (interquartile range (IQR) 49–59 years). The median body mass index value was 24.99 kg/m^2^ (IQR 22.48–29.04 kg/m^2^). The median size of the tumours was 1.55 cm (IQR 1.2–2.2 cm). All patients with cancer progression (*n* = 11) had a tumour diameter of above 2 cm. Forty-five patients (64.3%) were in post-menopausal status. The predominant histopathological subtype of breast tumour was invasive ductal carcinoma (IDC) at 84.3% of all studied patients, and 90.9% of the group had progression of the disease. Of the 70 women treated with adjuvant chemotherapy, 25 received anthracycline-containing drugs and 10 non-anthracycline therapy. None of the patients received neoadjuvant treatment.

The pre- and post-treatment AOPP concentrations were assessed according to undergone therapy procedures (Table 2). Interestingly, non-anthracycline therapy seemed to be associated with significantly elevated post-treatment AOPP concentration in comparison to patients who did not receive chemotherapy (*p* = 0.0086). Moreover, patients treated with non-anthracycline chemotherapy seem to have elevated concentrations of AOPPs during therapy, with the *p*-value close to statistical significance (*p* = 0.0996). The opposite observation in the AOPP level, also with closeness to statistical significance, was made for the group of patients not treated with any additional chemotherapy (*p* = 0.0778). However, tamoxifen administration seemed to decrease the post-treatment AOPP concentration in comparison to no adjuvant endocrine therapy (*p* = 0.0407). AOPP levels were found to increase during treatment among patients who were not treated with endocrine therapy (*p* = 0.0254). 

In Table 3, we present the pre- and post-treatment concentrations of AOPPs regarding some typical clinical features of breast tumour patients. The localisation of the malignancy in the left breast seems to be related to higher pre-treatment AOPP concentrations (*p* = 0.0056). However, there are no differences between the levels of AOPPs before and after the treatment according to the localisation of cancer (*p* > 0.05). In agreement with molecular subtypes of breast cancer, we found a significantly higher pre-treatment concentration of AOPPs in patients with luminal B HER2- than in those with other types (*p* < 0.0001). Interestingly, a significantly higher level of AOPPs was found in the group with luminal B HER2+ and non-luminal HER2+ than in the luminal A patients (*p* = 0.0053). After the treatment, a significantly lower concentration of AOPPs was shown in the luminal A breast cancer group than with other types (*p* < 0.0001). Our analysis shows that the level of AOPPs decreased during the treatment in the luminal A group of patients; however, it increased in the group with triple-negative cancer with *p* = 0.0102 and *p* = 0.0190, respectively. 

The conducted study also reveals that the tumour diameter slightly affects pre- (*p* = 0.0288) and strongly post-treatment (*p* = 0.0006) AOPP concentrations. Importantly, a tumour diameter < 2 cm is strongly related to a decreasing concentration of AOPPs during therapy (*p* = 0.0174). Nodal involvement and stage of disease seem to not have any impact on the level of measured oxidation protein products (*p* > 0.05). A tendency toward statistical significance was found for the group of women with grades 1 and 2 and related to the lower concentration of post-treatment AOPPs than in the group of patients with grade 3 according to the Elston and Ellis scale (*p* = 0.0890). The group of women with the IDC type of tumour demonstrated significantly higher values of pre-treatment AOPPs than the group with the invasive lobular carcinoma (ILC) type (*p* = 0.0115). 

To better understand the relationship between molecular biomarkers and the level of oxidative damage, the pre- and post-treatment concentrations of AOPPs were tested according to immunohistochemical markers of breast cancer (Table 4). It should be highlighted that overexpression of Ki-67 was related to elevated pre- and post-treatment levels of AOPPs (*p* = 0.0001 and *p* = 0.0041, respectively).

The status of the oestrogen receptor (ER) seems to have significant importance in the post-treatment concentration of AOPPs (*p* = 0.02) and be related to its increased value in the ER-negative group of breast cancer patients. A similar observation was also made for the expression of progesterone receptor (PR) (*p* = 0.0187). Additionally, the higher values of AOPPs after treatment in the ER-negative group of patients are also of considerable importance (*p* = 0.0360). The same relation is also shown by the group of PR-negative patients with a tendency to statistical significance (*p* = 0.0995). It is worth mentioning that both ER-positive and PR-positive groups are related to decreasing AOPP concentrations after therapy, with a tendency to statistical significance (*p* = 0.0617, *p* = 0.0739, respectively).

The human epidermal growth factor receptor 2 (HER2) and the status of E-cadherin seem to have no impact on the concentrations of AOPPs before and after undergoing procedures (*p* > 0.05).

### 3.2. The Dependence between AOPP Levels and Angiogenic Biomarkers

To assess the relationship between the level of the pre-treatment AOPPs on angiogenic biomarkers, breast cancer patients were divided into three subgroups according to pre-treatment AOPP levels below the 25th percentile (low), between the 25th and 75th percentile (moderate), and above the 75th percentile (high) (Table 5). This analysis showed that the concentration of pre-treatment VEGF-A significantly elevated with the increase in pre-treatment AOPP level (*p* = 0.0051). The concentration of pre-treatment sVEGFR1 was found to be the lowest for the group with pre-treatment AOPPs above 9.45 μM, with closeness to statistical significance (*p* = 0.0685).

In the next step, Spearman’s correlation analysis was performed to find associations between AOPP levels and concentrations of angiogenic biomarkers. The results illustrated in Table 6 indicate the positive correlation between pre-treatment AOPPs and the concentration of VEGF-A, which is a key mediator of angiogenesis (r = 0.2415, *p* = 0.0440). The pre-treatment sVEGFR1 concentration shows a tendency toward statistically significant negative relation with the post-treatment level of AOPPs (r = −0.2317, *p* = 0.0592). A statistically significant association between post-treatment AOPP levels and the concentration of sVEGFR2 was also found. Post-treatment AOPP concentration increases were related to a decrease in the value of sVEGFR2 after treatment (r = −0.3330, *p* = 0.0059). Post-treatment AOPP concentration also positively correlates with the level of AOPPs before treatment (r = 0.4103, *p* = 0.0006).

### 3.3. The Receiver Operating Characteristic (ROC) for Identifying Markers of Disease Progression

The next step of the analysis was constructing the ROC curves to identify the most accurate parameter in predicting cancer relapse and obtain the optimal cut-off values for the examined markers before as well as after treatment. The results received from the performed analysis are shown in Table 7 and Table 8.

The borderline of the diagnostic usefulness of the test was set according to the area under the ROC curve (AUC) ≥ 0.5 and *p* < 0.05. The conducted study of pre-treatment concentrations revealed that the highest level of predictiveness was found for AOPPs (AUC = 0.773, *p* = 0.0005). The ROC curve identified a serum AOPP concentration of 9.37 μM, with 78% sensitivity and 72.7% specificity, as the best cut-off value to predict the risk of cancer relapse. For this analysis, the other pre-treatment examined parameters showed no predictive value in our study group.

Interestingly, based on the ROC data for post-treatment concentrations of investigated markers, the AOPP value was also found to be the best marker for the prediction of cancer recurrence. The optimal cut-off value was 10.39 μM. The AUC was 0.86, *p*-value < 0.0001, with a sensitivity of 81.8%, and a specificity of 89.3%.

**Figure 2:**
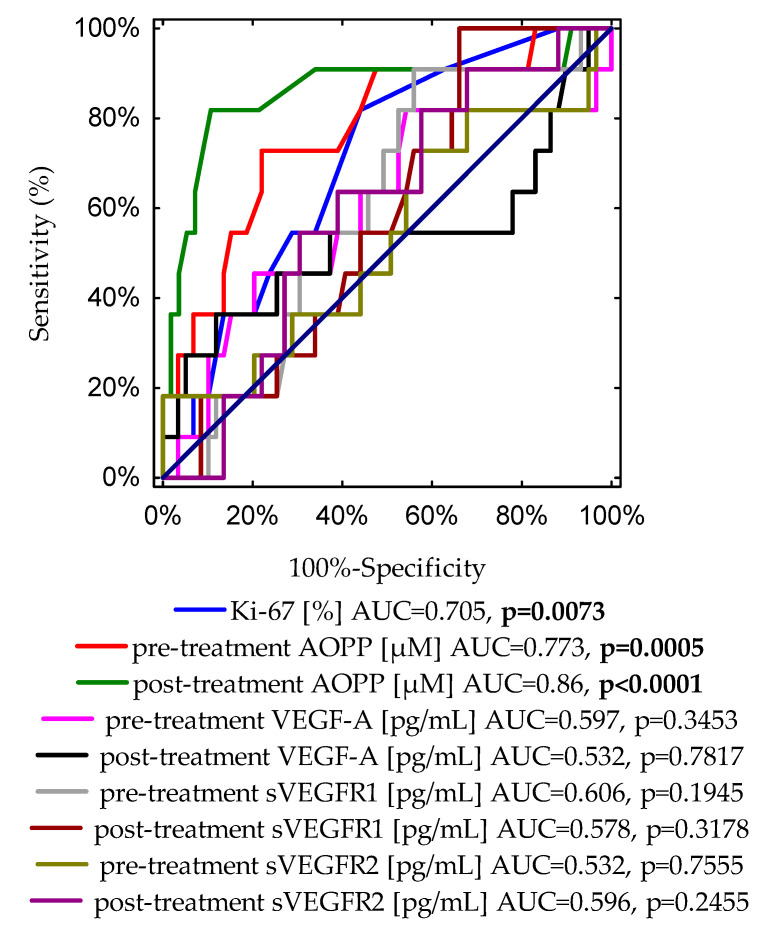
A graph showing nine ROC curves for the Ki-67 expression, pre- and post-treatment concentrations of AOPPs, VEGF-A, sVEGFR1 and sVEGFR2 with different values of area under the curve (AUC) and *p*-values for predicting disease-free survival.

Finally, Figure 2 demonstrates a graph with nine ROC curves for the Ki-67 expression, pre- and post-treatment concentrations of AOPP, VEGF-A, sVEGFR1, and sVEGFR2. The most accurate indicator for disease recurrence presents an area of 0.86 under the curve, which was reached for post-treatment AOPPs (*p* < 0.0001).

In addition, to compare the diagnostic accuracies of pre- and post-treatment AOPP concentrations, and typical prognostic biomarkers of disease recurrence, such as Ki-67 expression and tumour diameter, we conducted an additional ROC curve analysis (Figure S1). Importantly, the highest level of discrimination was found for the post-treatment AOPP concentration (AUC = 0.86, *p* < 0.0001). The receiver operating characteristic curve identified a post-treatment AOPP level of 10.39 μM, with 89.3% specificity and 81.8% sensitivity, as the best cut-off value to discriminate between relapse and non-relapse disease subjects. According to the ROC curve, cut-off points determined for other analysed parameters were as follows: for Ki-67 expression, the cut-off value was 15% with 55.9% specificity and 81.8% sensitivity (AUC = 0.705, *p* = 0.0073); the cut-off value for pre-treatment AOPP concentration was 9.37 μM with 78% specificity and 72.7% sensitivity (AUC = 0.773, *p* = 0.0005); for the tumour diameter, the cut-off point was 1.8 cm with 62.7% specificity and 81.8% sensitivity (AUC = 0.769, *p* = 0.0001).

### 3.4. Survival Analysis Regarding Pre- and Post-Treatment AOPP Concentrations

Subsequently, the cut-off points received from the ROC curve and the calculated medians for pre- and post-treatment AOPP concentrations were used to create Kaplan–Meier curves (Table 9). The study subjects were divided into two groups: below and above the cut-off points. The overall survival (OS) and disease-free survival (DFS) were calculated for each group.

**Figure 3:**
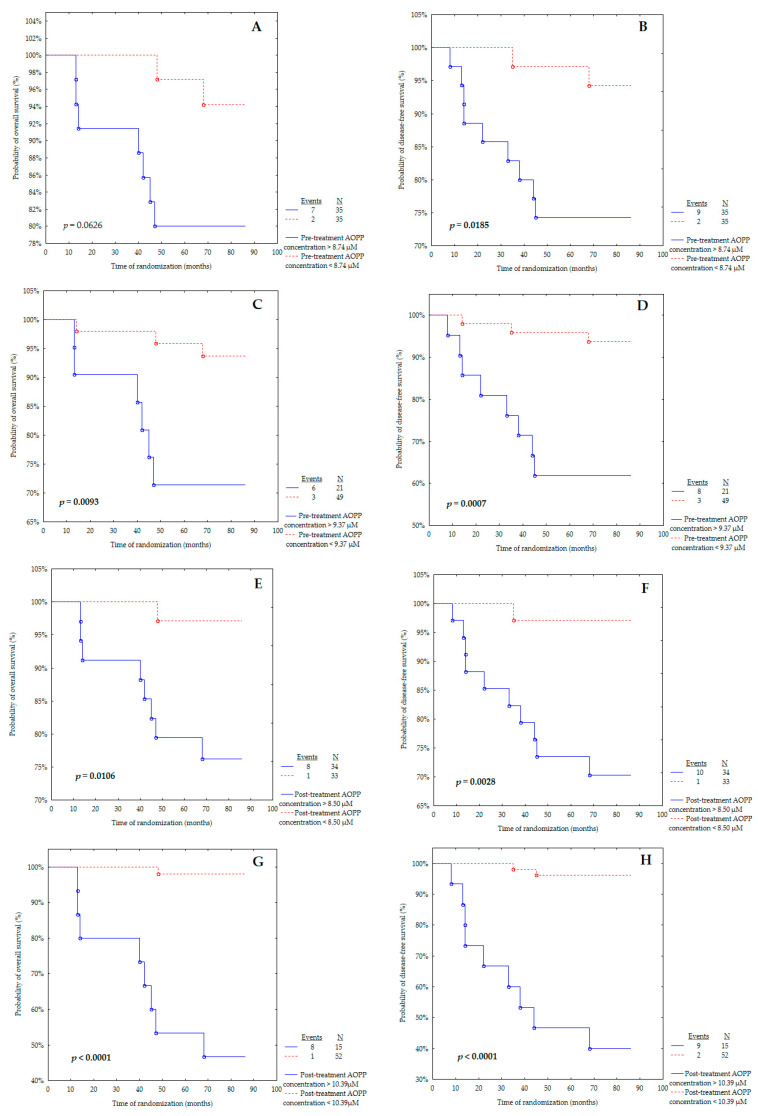
Kaplan–Meier curves for the overall survival (OS) and disease-free survival (DFS) analysis regarding (**A**,**B**) pre-treatment AOPP concentrations according to median; (**C**,**D**) pre-treatment AOPP concentrations according to ROC cut-off; (**E**,**F**) post-treatment AOPP concentrations according to median; (**G**,**H**) post-treatment AOPP concentrations according to ROC cut-off. Significant differences are marked by bold *p*-values.

For this analysis, almost all the determined cut-off values showed predictive value in our cohort (Figure 3A–H). Patients with a pre-treatment concentration of AOPPs above 9.37 μM had significantly worse OS than patients with AOPPs < 9.37 μM with *p* = 0.0093 (Figure 3C). However, the Kaplan–Meier curve determined according to the median of the pre-treatment level of AOPPs showed only closeness to statistical significance in the probability of OS (*p* = 0.0626) (Figure 3A). This observation discriminates against the use of the median as the ideal cut-off point and favours the use of the ROC cut-off point, which is much better at separating low-risk and high-risk patients for overall survival and cancer recurrence.

It is important to underline that the post-treatment AOPP concentration above 8.50 μM was related to poorer OS than the AOPP level below 8.50 μM with *p* = 0.0106 (Figure 3E). The same observation was made for the post-treatment AOPP concentration (Figure 3G), with the cut-off point obtained from the ROC curve (*p* < 0.0001).

Figure 3B,D presents the probability of disease-free survival for pre-treatment AOPP concentration according to the median or ROC cut-off point. The pre-treatment AOPP levels above 8.74 μM (Figure 3B) and above 9.37 μM (Figure 3D) were found and associated with a significantly increased probability of cancer relapse (*p* = 0.0185 and *p* = 0.0007, respectively).

Markedly, breast cancer patients with a post-treatment level of AOPPs above 8.50 μM were characterised as having lower DFS than the group with a concentration below 8.50 μM (Figure 3F) (*p* = 0.0028). A similar outcome was obtained for the post-treatment AOPP concentration (Figure 3H), with the cut-off point determined from the ROC curve (*p* < 0.0001).

Furthermore, the univariate Cox proportional hazards models of the disease-free survival and overall survival group (Table 10) were investigated to determine the risk score associated with biomarkers and to evaluate whether they could be independent prognostic factors for DFS or OS. The univariate analysis revealed that patients with AOPP levels below 8.74 μM before treatment have a significantly lower risk of shorter DFS compared to those with higher concentration (HR = 0.19, 95% CI 0.04–0.89, *p* = 0.0346). Moreover, a similar observation was also made for the post-treatment AOPP level; its decreased concentration was found to have a positive effect on the length of time without relapse of disease (HR = 0.08, 95% CI 0.01–0.66, *p* = 0.0185). The other investigated parameters showed no prognostic value on DFS in our cohort (*p* > 0.05).

Based on the results in Table 10, only the post-treatment AOPP concentration seems to have any impact on OS prediction. Its higher level, found in the univariate analysis, seems to be associated with poorer OS prognosis (HR = 0.11, 95% CI 0.01–0.86, *p* = 0.0358).

Furthermore, the multivariate Cox-proportional hazards models of the DFS and OS group, adjusted for age at the diagnosis, stage of the disease, nodal involvement, and expressions of ER, PR, HER2, and Ki-67 were investigated to determine the risk score associated with biomarkers and to evaluate whether they could be independent prognostic factors for DFS or OS (Table 11). The conducted analysis clearly shows that there is a strong link between the pre- and post-treatment levels of AOPPs and the risk of breast cancer relapse (*p* < 0.05). This study revealed that lower concentrations of AOPPs before and after treatment were positive predictors of long-term DFS (HR = 0.16, 95% CI 0.03–0.82, *p* = 0.0275; HR = 0.08, 95% CI 0.01–0.66, *p* = 0.0189, respectively). Data from the OS analysis indicate that an elevated post-treatment AOPP level, adjusted for the seven covariates (HR = 0.09, 95% CI 0.01–0.81, *p* = 0.0317), was associated with higher hazards of death. The investigation highlighted that pre- and post-treatment concentrations of AOPPs are independent prognostic factors for OS and DFS in breast cancer patients.

**Figure 4:**
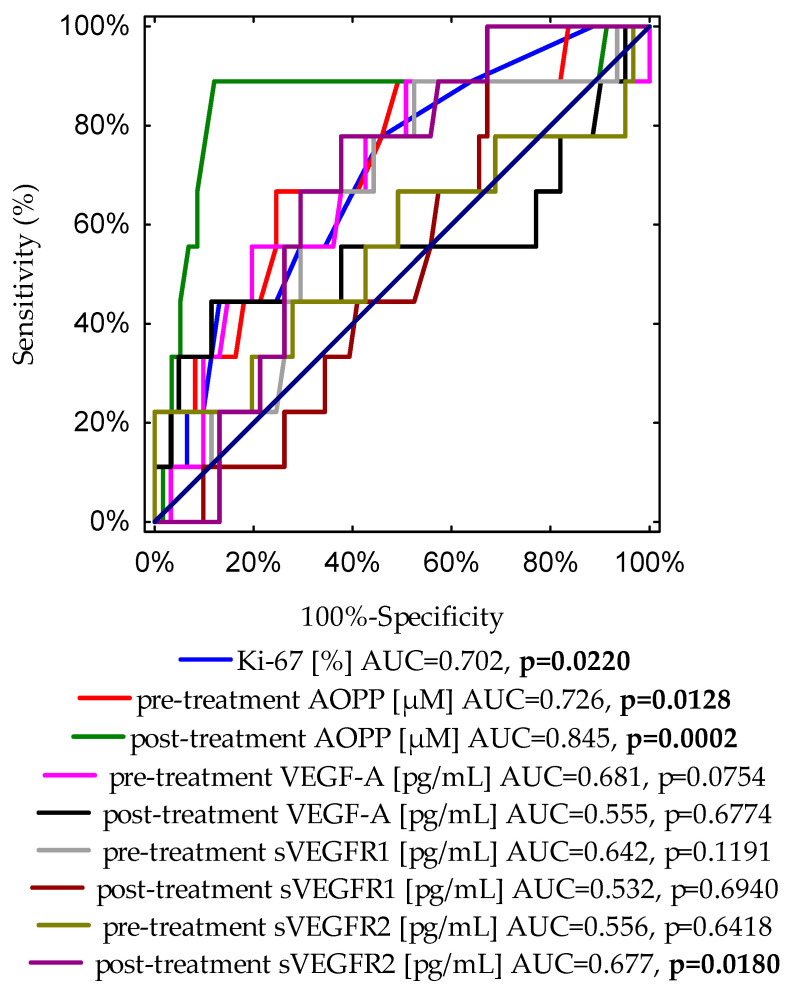
A graph showing nine ROC curves for Ki-67 expression, and pre- and post-treatment concentrations of AOPP, VEGF-A, sVEGFR1, and sVEGFR2 with different values of area under the curve (AUC) and *p*-values for predicting overall survival.

Additionally, we also evaluated the ROC curves to assess the diagnostic accuracies of the investigated variables for the prediction of overall survival in BrC subjects (Figure 4). This analysis indicates that in patients with BrC, next to Ki-67 expression, the cut-off value of 10.39 μM of AOPPs may predict the occurrence of death with 87.9% specificity and 88.9% sensitivity.

Moreover, we evaluated the ROC curve to assess the diagnostic accuracies of pre- and post-treatment AOPPs and frequently examined variables for the prediction of the BrC death incidence, such as Ki-67 expression and tumour diameter (Figure S2). It is noteworthy that the post-treatment AOPP cut-off value of 10.39 μM was found to be the best prognostic marker for the prediction of cancer-related death. Based on the ROC curve, cut-off points were provided for other determinants as follows: for Ki-67 expression, the cut-off value was 15% with 54.1% specificity and 77.8% sensitivity (AUC = 0.702, *p* = 0.0220); the cut-off value for pre-treatment AOPP concentration was 9.37 μM with 75.4% specificity and 66.7% sensitivity (AUC = 0.726, *p* = 0.0128); for the tumour diameter, the cut-off point was 2.1 cm with 70.5% specificity and 77.8% sensitivity (AUC = 0.773, *p* = 0.0005).

Table 12 shows the results of linear regression models for the occurrence of breast cancer relapse according to concentrations of the examined predictors. The recurrence of breast tumour was positively associated with pre-treatment AOPP concentration. Models 1, 2, 3, and 4, adjusted for included independent factors, show that elevated levels of pre-treatment AOPPs are strongly associated with cancer recurrence (*p* = 0.0018, *p* = 0.0007, *p* < 0.0001, and *p* = 0.0196, respectively). Additionally, regarding the concentration of AOPP after therapy is undergone, a similar observation was made. There was a significant linear positive relationship between post-treatment AOPP levels and the risk of disease relapse according to all models (*p* < 0.05).

Interestingly, after adjusting for covariates in Model 3, the negative effect of the soluble form of pre-treatment VEGF receptor type 1 was shown, i.e., its increase is related to extended DFS (*p* = 0.0198). Apart from this, Model 4, adjusted for age, BMI, parity, menopausal status, smoking status, tumour stage, tumour diameters, histological type, and nodal involvement, had a tendency toward statistical significance in a similar manner (*p* = 0.0593).

## 4. Discussion

Breast cancer, along with lung cancer, is the main cause of cancer-related deaths among women globally [1]. In the United States alone in 2023, it is estimated that about 297,790 women will be diagnosed with invasive breast cancer and about 43,700 women will die from breast cancer [52]. This clearly shows that it should be of great interest to find a useful marker, which could have a beneficial influence on the prediction of cancer-related events and give more information about the possible outcome of the patients in the early stage of the disease. The current study presents advanced AOPPs as a valuable biomarker for monitoring the course of BrC treatment and a predictor of cancer-related events.

AOPPs are dityrosine-containing and cross-linking protein products made as an effect of the reaction of albumin with chlorinated oxidants due to oxidative stress [53]. Significantly increased values of AOPPs in cancer patients compared to healthy subjects were found by some researchers [15,54,55,56,57,58,59]. The current study shows a positive correlation between pre-treatment AOPPs and VEGF-A, which could be evidence of a relationship between oxidative protein damage in breast cancer and excessive angiogenic process. The role of VEGF in pathological angiogenesis in malignant tumours is essential [60]. Interestingly, the current study shows that lower pre-treatment levels of sVEGFR1 and lower post-treatment levels of sVEGFR2 were related to elevated levels of post-treatment AOPPs. Elevated concentrations of AOPPs are associated with the intensification of neoplastic angiogenesis by increasing VEGF-A levels and reducing levels of sVEGFR1, which is a natural VEGF-A inhibitor. The interplay between the amount of oxidative damage enacted on proteins and angiogenic factors in cancerous disease is not fully understood, although it is suggested by Huang et al. that the VEGF signalling pathway is mainly controlled by the interactions between angiogenesis and oxidative stress [61]. Several studies have shown that the increased concentration of AOPP in diseases is associated with endothelial dysfunctions [17,62]. AOPPs serve mainly as an oxidative stress marker; however, some studies insinuate its role in the production of ROS, vascular inflammation, and a even pathogenic role in prostate cancer [22,57]. Huang et al. have observed the effect of AOPPs on the overexpression of VEGF and the elevated expression and secretion of sVEGFR1 in trophoblast cell lines. The authors have also suggested that AOPPs increase both the expression and secretion of VEGF in trophoblasts; however, they implicate that the increased secretion of sVEGFR1 overwhelms VEGF levels and leads to a reduction in VEGF’s biological effect. The authors indicate that AOPPs play a key role in mediating trophoblasts’ angiogenic regulation and signalling pathways [19]. However, Abd El-Khalik et al. have found a negative correlation between AOPPs and VEGF and a positive correlation between AOPPs and sVEGFR1 in patients with diabetic foot ulcers. The authors suggest that chronic inflammation in patients with diabetic foot ulcers, supported by the increased level of TNF-α, is associated with an enhanced production of sVEGFR1 and consequently a reduction in VEGF level [53]. 

Following this, we evaluated pre- and post-treatment AOPP concentrations according to treatment undergone as well as clinical, pathological, and molecular characteristics. Generally, in our cohort, the concentrations of AOPPs were decreased after the treatment procedures, which is in line with findings by Salehi et al. The authors indicated that the plasma AOPP levels decreased significantly in the colorectal cancer group 24 h after surgery. The authors suggest that the main culprit of the higher AOPP levels before surgery was the tumour [56]. On the contrary, Chiang et al. have observed a significantly higher concentration of plasma AOPPs four weeks after the resection of colorectal cancer compared to the level before surgery [63]. However, it is not known whether the findings by Chiang et al. can be associated with the persistent inflammatory status after resection of the tumour and/or with no information about the administration of additional therapy, which could potentially influence the oxidative status. Interestingly, an increase in AOPP levels after therapy was found in our group of patients not treated with adjuvant endocrine therapy. On the other hand, the administration of tamoxifen seemed to have the opposite effect on post-treatment AOPP concentrations, which were significantly decreased compared to the group not treated with endocrine therapy. This observation agrees with Ek et al.’s finding that tamoxifen administration is a part of antioxidant defence and can beneficially prevent oxidative stress-related diseases in post-menopausal women [64]. Additionally, in our cohort, patients who received non-anthracycline-containing drugs had significantly higher levels of post-treatment AOPPs compared to the group who did not receive chemotherapy. Silva et al. have reported that the administration of tamoxifen or chemotherapy or combined treatment to breast cancer patients is related to changes in the level of AOPPs before and after therapeutic procedures. The authors found that the AOPP levels after treatment were significantly elevated in the group of subjects treated only with chemotherapy compared to those who received only tamoxifen or chemotherapy, followed by tamoxifen, and had a tendency to increase during the treatment. It is insisted by the authors that chemotherapy alone has a deleterious effect on the balance between the oxidant and antioxidant systems [65]. It is also suggested that some of the clinical and pathological features of breast cancer subjects may reflect the high risk of poor outcomes. In our study, AOPP levels decreased significantly after treatment procedures; however, in patients with triple-negative breast cancer (ER-/PR-/HER2-), AOPP levels increased after the therapy. It should be noted that the triple-negative type of cancer is associated with a more aggressive clinical course, early metastasis and poor outcome [66]. An opposite observation was made for the group with luminal A, as AOPP levels were significantly lower after therapy, which is in line with the good prognosis and longer OS reported by some researchers [67,68]. It is well known that a left-sided tumour is considered to be more aggressive than a right-sided one and is associated with poorer outcomes [69]. The results of our study show that patients with a localization of the tumour on the left side had a significantly higher level of pre-treatment AOPPs; however, the localization of the tumour did not significantly affect the patients’ future conditions. Additionally, the results of our study show that the patients with a tumour size above 2 cm (T2 stage according to TNM classification) had significantly higher values of pre- and post-treatment AOPP concentrations than patients with a diameter <2 cm (Table 3), and larger tumour size is assumed to be a high-risk clinical feature of developing of recurrence [70]. Surprisingly, Salehi et al. did not find any significant associations between AOPP levels and both tumour diameter and TNM stage in colorectal cancer patients [56]. Due to the limited literature data, further research is needed to reach a consensus on this. In addition, the group of patients with a Ki-67 index above 20% had significantly elevated levels of pre- and post-treatment AOPPs, which indicate a higher risk of breast cancer relapse in our cohort. Nelson et al. have linked the expression of Ki-67 above 20% to a higher incidence of cancer recurrence, which is in agreement with our findings [70].

According to our linear regression results, it can be noted, regardless of the model used, that higher pre- and post-treatment AOPP concentrations are positively correlated with a higher risk of cancer recurrence. Zhou et al. have assessed the levels of AOPPs in acute myeloid leukaemia patients and observed their tendency to increase during the progression of cancer. The authors conclude that there is a strong association between persisting oxidative damage and a higher risk of acute myeloid leukaemia relapse, which suggests that oxidative stress might be a key factor in cancer progression [71]. In contrast, plasma AOPPs were found by Delrieu et al. to be a favourable factor in metastatic breast cancer patients undergoing 6 months of physical activity intervention. Patients with cancer progression or who died during 6-month follow-up had significantly lower concentrations of AOPPs. However, a reduction in AOPP concentration may have been associated with the elevated antioxidant efficiency induced by regular physical activity in this group [72]. It should be emphasized that our ROC analysis demonstrates that pre- and post-treatment AOPP levels in serum have a high diagnostic value in differentiating the groups of early-stage women with or without breast cancer recurrence in our cohort, with sensitivities at 78%, 81.8% and specificities at 72.7%, 89.3%, respectively. Figure 2 and Figure S1 clearly emphasize the strength of post-treatment AOPP concentration (AUC = 0.86, *p* < 0.0001) as a marker for estimating the likely course of the disease. Due to the higher sensitivity (81.8%) and specificity (89.3%) of AOPP concentration after treatment and the easy-to-perform and economical measurement procedure, AOPP concentration may compete with Ki-67 expression (sensitivity 81.8%, specificity 55.9%; AUC = 0.705, *p* = 0.0073) as a prognostic marker for the development of cancer recurrence. Our findings indicate the relevant usefulness of AOPP measurements in the differentiation between relapse and non-relapse breast cancer patients. Kundaktepe et al. showed no predictive value of AOPPs in separating breast cancer patients with or without metastases. However, this study included BrC patients with distant metastases at the time of the diagnosis [54]. In the study by Mahmoud et al., increased AOPP levels were found in acute lymphoblastic leukaemia patients compared to the controls, and their tendency to increase after the administration of chemotherapy was observed; however, the Kaplan–Meier analysis showed no association between AOPP concentrations and the risk of cancer relapse. This finding might probably be related to the small number of patients included in this study [73]. Several studies have discussed the possible use of AOPPs as a marker for the prediction of various types of disease or outcomes in patients. Kaneda et al. have reported that the higher concentrations of AOPPs observed in the plasma of coronary artery disease patients were related to an increased incidence rate and severity in these patients [74]. According to the results of Bagyura et al., AOPPs seem to have possible predictive value as an early marker of atherosclerosis in men. It is implicated that AOPPs, through various mechanisms, lead to endothelial dysfunction and eventually to the development of atherosclerosis [17]. In addition, the results of the logistic regression conducted by Koike et al. suggest that AOPPs may serve as a predictive factor in suspicious digital rectal examinations [57]. No previous studies have shown the prognostic value of higher AOPP concentrations on breast cancer relapse in the early stage. Perhaps serum AOPPs should be taken into consideration by clinicians as a sensitive marker in the monitoring of breast cancer recurrence.

The results of our univariate Cox regression analysis suggest that the lower levels of pre- and post-treatment AOPPs may serve as a prognostic factor of better and prolonged OS. This is also supported by the results of the multivariate Cox regression when adjusted for age at the diagnosis, stage of the disease, nodal involvement, and expression of ER, PR, HER2, and Ki-67. Both the pre- and post-treatment lower levels of AOPPs were positive predictors of long-term overall survival in our cohort. Our results indicate that pre- and post-treatment concentrations of AOPPs above the cut-off points (9.37 μM and 10.39 μM, respectively) were associated with a shorter OS time in our study group. The high sensitivity (88.9%) and specificity (87.9%) of AOPP concentrations after treatment compared with the sensitivity (77.8%) and specificity (54.1%) of Ki-67 expressions suggest that AOPPs may constitute an additional, useful, and easy-to-perform prognostic marker of unfavourable overall survival. Based on a series of statistical analyses (ROC curves, Kaplan–Meier method, uni- and multivariate Cox regression), our study suggests that in patients with BrC, AOPPs may compete with Ki-67 as a prognostic marker of cancer-related death. This is the first study showing that higher AOPP levels in early-stage breast cancer patients are related to poorer OS. Furthermore, several researchers have linked higher levels of AOPPs to increased mortality in various states. The multivariate Cox analysis conducted by Cai et al., which was performed for a group of patients with diagnosed hepatitis B virus-related acute-on-chronic liver failure, revealed that AOPPs can serve as an independent prognostic factor of early mortality [75]. In addition, Zhou et al. assessed the levels of AOPPs in patients who were undergoing maintenance haemodialysis. The authors observed that higher levels of AOPPs are associated with an elevated risk of all-cause mortality in their cohort. The authors concluded that AOPP concentrations may serve as a relevant risk marker for the early detection of mortality in maintenance haemodialysis patients [21]. Moreover, Suvakov et al. have investigated AOPP levels in patients with end-stage renal disease and found that higher plasma AOPP levels were related to higher cardiovascular-specific mortality. Due to the role of AOPPs in endothelial dysfunction, the authors suggest including oxidative biomarkers in diagnostic panels for better monitoring of end-stage renal patients and to improve the choice of appropriate therapeutic patterns [62]. The study conducted by Elkabany et al. shows that a higher level of AOPPs is associated with the severity of respiratory distress syndrome in neonates. The ROC curve analysis conducted by the authors also suggests that the cut-off value of AOPPs can be useful in predicting the severity and high risk of mortality in neonatal respiratory distress syndrome [76]. It should be highlighted that the ability to estimate the prognosis of patients with BrC depending on the pre- and post-treatment concentrations of AOPPs may be beneficial for assessing a patient’s future condition and life quality. 

The findings should be interpreted with caution due to the following limitations of this study. The results were from a relatively small number of patients in a single centre. A total of 22 patients who underwent BCS or mastectomy were excluded due to their ineligibility for further prognostic analysis (Figure 1). The study was performed in a daily clinical routine, the sample size was dependent on receiving the patients’ consent for participation, and very restrictive inclusion and exclusion criteria also influenced the limited number of patients in the project. In addition, we recruited patients of Polish descent only, so our results may not apply to other ethnic groups. We recruited patients at an early stage of BrC without metastases, so we cannot determine what the prognostic value would be for larger and more advanced tumours. A larger sample size of patients will be advantageous to the validation of long-term effects. Another limitation was the lack of a control group, and further research with a group of healthy subjects should be conducted.

Nevertheless, AOPP concentrations may serve as potential prognostic candidates because the method of measuring their concentration is inexpensive and easy to perform. Additionally, further research may provide new possibilities for the prevention and treatment of neoplastic diseases based on reducing their concentration or preventing the formation of AOPPs.

## 5. Conclusions

Our results suggest that both pre-treatment AOPP levels above 9.37 μM (sensitivity: 78%, specificity: 72.7%) and post-treatment concentrations of AOPPs above 10.39 μM (sensitivity: 81.8%, specificity: 89.3%) may promote the probability of recurrence in BrC patients. Additionally, Kaplan–Meier survival analysis shows that pre- and post-treatment AOPP concentrations above 9.37 μM and 10.39 μM, respectively, may increase the likelihood of cancer-related death in the BrC cohort. An absence of administration of endocrine drugs seems to increase the level of AOPPs after treatment procedures. In addition, a positive relation between AOPPs and VEGF-A was found, and a negative relationship between AOPPs and sVEGFR1 and sVEGFR2 was revealed, which clearly supports the involvement of AOPPs in neoplastic angiogenesis. Based on numerous types of statistical analyses, it seems that AOPPs are a better prognostic biomarker than VEGF-A, although the current study confirmed the strong relationship between AOPPs and angiogenic parameters. It would seem that the findings of our study are promising and relevant for further research in a larger patient population with invasive breast cancer.

## Figures and Tables

**Table 1:** Baseline characteristics of women with breast cancer.

Demographic and Clinical Data	Overall (*n* = 70)	Patients without Progression (*n* = 59)	Patients with Progression (*n* = 11)
	n (%)		
Age (according to median)			
<54 years	32 (45.7%)	26 (44.1%)	6 (54.5%)
≥54 years	38 (54.3%)	33 (55.9%)	5 (45.5%)
Menopausal status			
Pre-menopausal	25 (35.7%)	19 (32.2%)	6 (54.5%)
Post-menopausal	45 (64.3%)	40 (67.8%)	5 (45.5%)
BMI (kg/m^2^)			
Normal (18.5 ≤ 24.99)	34 (48.6%)	26 (44.1%)	8 (72.7%)
Overweight (25 ≤ 29.99)	22 (31.4%)	21 (35.6%)	1 (9.1%)
Obese (>30)	14 (20%)	12 (20.3%)	2 (18.2%)
Parity status			
0	6 (8.6%)	3 (5.1%)	3 (27.3%)
1–2	49 (70%)	44 74.6%)	5 (45.5%)
3 and more	15 (21.4%)	12 (20.3%)	3 (27.3%)
Localization of tumour			
Right breast	35 (50%)	29 (49.2%)	6 (54.5%)
Left breast	35 (50%)	30 (50.8%)	5 (45.5%)
Diameter of the tumour			
T1 < 2 cm	45 (64.3%)	45 (76.3%)	0 (0%)
2 cm < T2 < 5 cm	25 (35.7%)	14 (23.7%)	11 (100%)
Lymph node status			
N0	51 (72.9%)	45 (76.3%)	6 (54.5%)
N1	19 (27.1%)	14 (23.7%)	5 (45.5%)
Histological type			
IDC	59 (72.9%)	49 (83.1%)	10 (90.9%)
ILC	11 (27.1%)	10 (16.9%)	1 (9.1%)
Grade according to Elston-Ellis			
1 + 2	53 (75.7%)	45 (76.3%)	8 (72.7%)
3	17 (24.3%)	14 (23.7%)	3 (27.3%)
Molecular type			
Luminal A (HR+/HER2−/Ki-67 < 20%)	41 (58.6%)	38 (64.4%)	3 (27.3%)
Luminal B (HR+/HER2−/Ki-67 ≥ 20%)	14 (20%)	9 (15.3%)	5 (45.5%)
Luminal B HER2+ (HR+ HER2+)	7 (10%)	6 (8.6%)	1 (9.1%)
Triple negative (HR-/HER2-)	8 (11.4%)	6 (8.6%)	2 (18.2%)
Staging			
I	31 (44.3%)	30 (50.8%)	1 (9.1%)
II	39 (55.7%)	29 (49.2%)	10 (90.9%)
Progesterone receptor (PR)			
Negative	16 (22.9%)	13 (22%)	3 (27.3%)
Positive	54 (77.1%)	46 (78%)	8 (72.7%)
Oestrogen receptor (ER)			
Negative	11 (27.1%)	9 (15.3%)	2 (18.2%)
Positive	59 (72.9%)	50 (84.7%)	9 (81.8%)
E-cadherin			
Negative	5 (7.1%)	5 (8.5%)	0 (0%)
Positive	65 (92.9%)	54 (91.5%)	11 (100%)
Ki-67			5 (45.5%)
<20%	43 (61.4%)	38 (64.6%)	6 (54.5%)
≥20%	27 (38.6%)	21 (35.6%)	
Comorbidities			
Heart attack	3 (4.3%)	3 (5.1%)	0 (0%)
Hypertension	17 (24.3%)	16 (27.1%)	1 (9.1%)
Diabetes	1 (1.4%)	1 (1.7%)	0 (0%)

Abbreviations: BMI: body mass index; N0: no evidence of spread to lymph nodes; N1: spread to axillary lymph nodes; IDC: invasive ductal carcinoma; ILC: invasive lobular carcinoma; HR+: hormone receptor positive; HR-: hormone receptor negative; HER2-: human epidermal growth factor receptor 2 negative; HER2+: human epidermal growth factor receptor 2 positive; Ki-67: marker of proliferation Ki-67.

**Table 2:** Levels of AOPPs in breast cancer patients before and after treatment.

Feature/ Number of Patients (%)	Pre-Treatment AOPP Concentration [µM] *n* = 70	Post-Treatment AOPP Concentration [µM] *n* = 67 ^#^	Pre-Treatment vs. Post-Treatment AOPPs *n* = 67 ^#^ *p*-Value
Surgery	*p* = 0.5737	*p* = 0.8831	
BCS + Radiotherapy *n* = 58/55 (82.9%/82.1%)	8.69 (1.17)	8.40 (2.44)	0.3260
Mastectomy *n* = 12/12 (17.1%/17.9%)	8.89 (0.78)	8.52 (2.72)	0.6745
Chemotherapy	*p* = 0.3471	***p* = ** **0.0283**	
Anthracycline *n* = 25/24 (35.7%/35.8%)	8.84 (1.07)	8.59 (2.29)	0.6074
Non-anthracycline *n* = 10/9 (14.3%/13.4%)	9.06 (0.86)	10.24 * (1.25)	0.0996
No *n* = 35/34 (50%/50.7%)	8.54 (1.19)	7.82 (2.63)	0.0778
Endocrine therapy	*p* = 0.9639	*p* = 0.3461	
Tamoxifen *n* = 35/34 (50%/50.7%)	8.70 (1.06)	8.08 ** (2.94)	0.1910
Inhibitor aromatase *n* = 13/13 (18.6%/19.4%)	8.68 (1.35)	8.17 (2.09)	0.2273
Tamoxifen and inhibitor aromatase *n* = 8/8 (11.4%/11.9%)	9.00 (1.10)	8.36 (1.66)	0.1577
Other type *n* = 2/2 (2.9%/3%)	8.54 (0.95)	8.50 (0.45)	0.9745
No *n* = 12/10 (17.1%/14.9%)	8.70 (1.16)	9.93 (1.46)	**0.0254**
Brachytherapy	*p* = 0.6461	*p* = 0.5656	
Yes *n* = 36/36 (51.4%/53.7%)	8.67 (1.23)	8.58 (2.44)	0.8275
No *n* = 34/31 (48.6%/46.3%)	8.79 (0.97)	8.23 (2.53)	0.1815

Abbreviations: AOPP: advanced oxidation protein products; BCS: breast-conserving surgery. Data are expressed as a mean and standard deviation (SD). Significant differences are denoted in bold. The underlined *p*-values represent closeness to statistical significance.* *p* < 0.0086 vs. No chemotherapy; ** *p* < 0.0407 vs. No endocrine therapy; **^#^** The post-treatment AOPP measurements included 67 subjects due to a lack of biological samples from three women.

**Table 3:** AOPP levels in respect to clinical and pathological characteristics in breast cancer subjects.

Feature	Number of Patients (%)	Pre-Treatment AOPP Concentration [μM] *n* = 70	Post-Treatment AOPP Concentration [μM] *n* = 67 ^#^	Pre-Treatment vs. Post-Treatment AOPPs *n* = 67 ^#^ *p*-Value
Tumour localisation		***p* = ** **0.0056**	*p* = 0.5014	
Left breast	35/33 (50%/49.3%)	9.09 (1.09)	8.63 (2.19)	0.1685
Right breast	35/34 (50%/50.7%)	8.36 (1.02)	8.22 (2.74)	0.7493
Molecular subtypes		***p*** **< 0.0001**	***p*** **< 0.0001**	
Luminal A	41/40 (58.6%/59.7%)	8.23 (0.79)	7.27 *** (2.18)	**0.0102**
Luminal B HER2-	14/14 (20%/20.9%)	10.05 * (0.86)	10.20 (2.29)	0.7808
Luminal B HER2+ and Non-Luminal HER2+	7/7 (10%/10.4%)	9.24 ** (1.03)	9.75 (0.98)	0.4711
Triple negative	8/6 (11.4%/9%)	8.50 (1.01)	10.32 (1.39)	**0.0190**
Tumour diameter		***p* = ** **0.0288**	***p* = ** **0.0006**	
T1 2 cm	45/43 (64.3%/64.2%)	8.51 (1.03)	7.68 (2.06)	**0.0174**
2 cm < T2 < 5 cm	25/24 (35.7%/35.8%)	9.11 (1.16)	9.76 (2.61)	0.1671
Nodal status		*p* = 0.8878	*p* = 0.5610	
N0	51/48 (72.9%/71.6%)	8.74 (1.13)	8.53 (2.33)	0.5414
N1	19/19 (27.1%/28.4%)	8.69 (1.09)	8.14 (2.84)	0.3264
Stage of disease		*p* = 0.1465	*p* = 0.2139	
IA	31/29 (44.3%/43.3%)	8.51 (1.11)	7.99 (1.91)	0.2416
IIA + IIB	39/38 (55.7%/56.7%)	8.90 (1.09)	8.75 (2.80)	0.6761
Elston and Ellis grade		*p* = 0.2782	*p* = 0.0890	
G1 + G2	53/51 (75.7%/76.1%)	8.64 (1.20)	8.13 (2.50)	0.1016
G3	17/16 (24.3%/23.9%)	8.98 (0.72)	9.34 (2.17)	0.4938
Histological type		***p* = ** **0.0115**	*p* = 0.1471	
IDC	59/56 (84.3%/83.6%)	8.87 (1.12)	8.62 (2.45)	0.4067
ILC	11/11 (15.7%/16.4%)	7.96 (0.65)	7.43 (2.46)	0.4581

Abbreviations: AOPP: advanced oxidation protein products; HER2-: human epidermal growth factor receptor 2 negative; HER2+: human epidermal growth factor receptor 2 positive; N0: no evidence of spread to lymph nodes; N1: spread to axillary lymph nodes; IDC: invasive ductal carcinoma; ILC: invasive lobular carcinoma. Data are expressed as a mean and standard deviation (SD). Significant differences are denoted by bold. The underlined *p*-values represent closeness to statistical significance; * *p* < 0.0001 vs. Luminal A, *p* = 0.0001 vs. Triple negative; *p* = 0.0427 vs. Luminal B HER2+ and Non-Luminal HER2+; ** *p* = 0.0053 vs. Luminal A; *** *p* < 0.0001 vs. Luminal B HER2-; *p* = 0.0006 vs. Luminal B HER2+ and Non-Luminal HER2; *p* = 0.008 vs. Triple negative. **^#^** The post-treatment AOPP measurements included 67 subjects due to a lack of biological samples from three women.

**Table 4:** The AOPP concentrations according to molecular characteristics in breast cancer cases.

Feature	Number of Patients (%)	Pre-Treatment AOPP Concentration [μM] *n* = 70	Post-Treatment AOPP Concentration [μM] *n* = 67 ^#^	Pre-Treatment vs. Post-Treatment AOPPs *n* = 67 ^#^ *p*-Value
Expression of Ki-67		***p* = ** **0.0001**	***p* = ** **0.0041**	
<20%	43/42 (61.4%/62.7%)	8.35 (0.89)	7.79 (2.64)	0.1748
≥20%	27/25 (38.6%/37.3%)	9.36 (1.17)	9.56 (1.63)	0.7258
Expression of HER2		*p* = 0.2022	*p* = 0.1688	
Positive	7/6 (10%/9%)	9.24 (1.03)	9.75 (0.98)	0.1918
Negative	63/61 (90%/91%)	8.67 (1.11)	8.29 (2.54)	0.4711
Hormone receptor status				
ER+	59/58 (84.3%/86.6%)	*p* = 0.6436 8.70 (1.12)	***p* = 0.0200** 8.15 (2.50)	0.0617
ER-	11/9 (15.7%/13.4%	8.87 (1.06)	10.19 (1.29)	**0.0360**
PR+	54/53 (77.1%/79.1%)	*p* = 0.2128 8.64 (1.15)	***p* = 0.0187** 8.06 (2.59)	0.0739
PR-	16/14 (22.9%/20.9%)	9.03 (0.91)	9.79 (1.29)	0.0995
E-cadherin status		*p* = 0.0862	*p* = 0.2403	
Positive	65/62 (92.9%/92.5%)	8.79 (1.11)	8.52 (2.44)	0.3643
Negative	5/5 (7.1%/7.5%)	7.91 (0.74)	7.17 (2.80)	0.5055

Abbreviations: AOPP: advanced oxidation protein products; Ki-67, proliferation marker; HER, human epidermal growth factor receptor 2; ER, oestrogen receptor; PR, progesterone receptor. Data are expressed as a mean and standard deviation (SD). Significant differences are denoted by bold. An underlined *p*-value represents closeness to statistical significance. ^#^ The post-treatment AOPP measurements included 67 subjects due to a lack of biological samples from three women.

**Table 5:** Pre-treatment levels of angiogenic biomarkers according to pre-treatment AOPP concentrations.

Parameter [units]	AOPP Low (<7.87 μM) *n* = 12	AOPP Moderate (7.87–9.45 μM) *n* = 41	AOPP High (>9.45 μM) *n* = 17	*p*-Value
VEGF-A concentration [pg/mL]	36.78	64.87	74.12	**0.0051**
sVEGFR1 concentration [pg/mL]	80.82	30.29	24.45	0.0685
sVEGFR2 concentration [pg/mL]	8468.45	9778.25	9182.05	0.8836

Abbreviations: AOPP: advanced oxidation protein products; VEGF-A: vascular endothelial growth factor A; sVEGFR1: soluble form of vascular endothelial growth factor receptor type 1; sVEGFR2: soluble form of vascular endothelial growth factor receptor type 2. Significant differences are denoted by bold *p*-values. An underlined *p*-value represents closeness to statistical significance.

**Table 6:** Spearman’s correlation analysis between AOPPs and angiogenic factors.

Parameters [Concentration]	AOPP			
	Pre-Treatment		Post-Treatment	
	r	*p*	r	*p*
Pre-treatment AOPPs [μM]	---	---	**0.4103**	**0.0006**
Post-treatment AOPPs [μM]	**0.4103**	**0.0006**	---	---
Pre-treatment VEGF-A [pg/mL]	**0.2415**	**0.0440**	−0.0668	0.5914
Post-treatment VEGF-A [pg/mL]	0.1253	0.3013	0.1574	0.2033
Pre-treatment sVEGFR1 [pg/mL]	−0.1061	0.3822	−0.2317	0.0592
Post-treatment sVEGFR1 [pg/mL]	−0.0498	0.6822	−0.1085	0.3821
Pre-treatment sVEGFR2 [pg/mL]	−0.0369	0.7615	0.0128	0.9184
Post-treatment sVEGFR2 [pg/mL]	−0.1770	0.1426	−**0.3330**	**0.0059**

Abbreviations: AOPP: advanced oxidation protein products; VEGF-A: vascular endothelial growth factor A; sVEGFR1: soluble form of vascular endothelial growth factor receptor type 1; sVEGFR2: soluble form of vascular endothelial growth factor receptor type 2; r: Spearman’s correlation coefficient; significant differences are denoted by bold *p*-values. An underlined *p*-value represents closeness to statistical significance.

**Table 7:** Results of predictive accuracy for pre-treatment AOPPs, VEGF-A, sVEGFR1, and sVEGFR2.

ROC Data	Pre-Treatment AOPP Concentration	Pre-Treatment VEGF-A Concentration	Pre-Treatment sVEGFR1 Concentration	Pre-Treatment sVEGFR2 Concentration
AUC	0.773	0.597	0.606	0.532
Youden index	0.51	0.28	0.35	0.18
Cut-off point	9.37	74.12	37.81	4626.99
Sensitivity (%)	78	81.8	90.9	18.2
Specificity (%)	72.7	45.8	44.1	100.0
Positive predictive Value (%)	38.1	22.0	23.3	100.0
Negative predictive Value (%)	93.9	93.1	96.3	86.8
Accuracy (%)	77.1	51.4	51.4	87.1
*p*-Value	**0.0005**	0.3453	0.1945	0.7555

Abbreviations: AOPP: advanced oxidation protein products; VEGF-A: vascular endothelial growth factor A; sVEGFR1: soluble form of vascular endothelial growth factor receptor type 1; sVEGFR2: soluble form of vascular endothelial growth factor receptor type 2; AUC: area under the curve. Significant differences are denoted by bold *p*-values.

**Table 8:** Results of predictive accuracy for post-treatment AOPPs, VEGF-A, sVEGFR1, sVEGFR2.

ROC Data	Post-Treatment AOPP Concentration	Post-Treatment VEGF-A Concentration	Post-Treatment sVEGFR1 Concentration	Post-Treatment sVEGFR2 Concentration
AUC	0.86	0.532	0.578	0.596
Youden index	0.71	0.24	0.34	0.25
Cut-off point	10.39	39.92	386.5	7230.0
Sensitivity (%)	81.8	36.4	100.0	63.6
Specificity (%)	89.3	88.1	33.9	61.0
Positive predictive Value (%)	60.0	36.4	22.0	23.3
Negative predictive Value (%)	96.2	88.1	100.0	90.0
Accuracy (%)	88.1	80.0	44.3	61.4
*p*-Value	**<0.0001**	0.7817	0.3178	0.2455

Abbreviations: AOPP: advanced oxidation protein products; VEGF-A: vascular endothelial growth factor A; sVEGFR1: soluble form of vascular endothelial growth factor receptor type 1; sVEGFR2: soluble form of vascular endothelial growth factor receptor type 2; AUC: area under the curve. Significant differences are denoted by bold *p*-values.

**Table 9:** Calculated medians and ROC cut-off point values of AOPPs before and after treatment.

	Pre-Treatment AOPP Concentration (μM)	Post-Treatment AOPP Concentration (μM)
Medians	8.74	8.50
ROC cut-off points	9.37	10.39

**Table 10:** Univariate analysis (Cox regression) of pre- and post-treatment levels of variables in relation to disease-free survival (DFS) and overall survival (OS).

	Univariate					
	DFS			OS		
Variables	HR	95% CI	*p*-Value	HR	95% CI	*p*-Value
Pre-treatment AOPP concentration						
Low vs. High	0.19	(0.04–0.89)	**0.0346**	0.25	(0.05–1.21)	0.0847
Post-treatment AOPP concentration						
Low vs. High	0.08	(0.01–0.66)	**0.0185**	0.11	(0.01–0.86)	**0.0358**
Pre-treatment VEGF-A concentration						
Low vs. High	1.89	(0.55–6.46)	0.3101	3.85	(0.80–18.56)	0.0924
Post-treatment VEGF-A concentration						
Low vs. High	1.21	(0.37–3.96)	0.7552	1.28	(0.34–4.78)	0.7122
Pre-treatment sVEGFR1 concentration						
Low vs. High	1.89	(0.55–6.46)	0.3102	3.86	(0.80–18.60)	0.0921
Post-treatment sVEGFR1 concentration						
Low vs. High	1.26	(0.38–4.13)	0.7015	0.83	(0.22–3.11)	0.7873
Pre-treatment sVEGFR2 concentration						
Low vs. High	0.85	(0.26–2.79)	0.7905	1.29	(0.35–4.80)	0.7057
Post-treatment sVEGFR2 concentration						
Low vs. High	1.87	(0.55–6.39)	0.3181	3.76	(0.78–18.09)	0.0988

Abbreviations: DFS: disease-free survival; OS: overall survival; HR: hazard ratio; CI: Confidence interval; AOPP: advanced oxidation protein products; VEGF-A: vascular endothelial growth factor A; sVEGFR1: soluble form of vascular endothelial growth factor receptor type 1; sVEGFR2: soluble form of vascular endothelial growth factor receptor type 2. Significant differences are marked by bold *p*-values.

**Table 11:** Multivariate analysis (Cox regression) of pre- and post-treatment levels of variables in relation to disease-free survival (DFS) and overall survival (OS).

	Multivariate					
	DFS			OS		
Variables	HR	95% CI	*p*-Value	HR	95% CI	*p*-Value
Pre-treatment AOPP concentration						
Low vs. High	0.16	(0.03–0.82)	**0.028**	0.2	(0.04–1.10)	0.065
Post-treatment AOPP concentration						
Low vs. High	0.08	(0.01–0.66)	**0.019**	0.09	(0.01–0.81)	**0.031**
Pre-treatment VEGF-A concentration						
Low vs. High	1.45	(0.39–5.31)	0.578	2.81	(0.54–14.70)	0.22
Post-treatment VEGF-A concentration						
Low vs. High	0.48	(0.07–3.26)	0.45	0.63	(0.07–5.37)	0.67
Pre-treatment sVEGFR1 concentration						
Low vs. High	1.51	(0.41–5.59)	0.537	2.89	(0.56–15.04)	0.207
Post-treatment sVEGFR1 concentration						
Low vs. High	0.91	(0.25–3.38)	0.893	0.66	(0.16–2.77)	0.568
Pre-treatment sVEGFR2 concentration						
Low vs. High	0.38	(0.08–1.90)	0.239	0.6	(0.11–3.42)	0.568
Post-treatment sVEGFR2 concentration						
Low vs. High	2.74	(0.73–10.18)	0.133	7.35	(1.16–46.77)	**0.035**

Abbreviations: disease-free survival; OS: overall survival; HR: hazard ratio; CI: Confidence interval; AOPP: advanced oxidation protein products; VEGF-A: vascular endothelial growth factor A; sVEGFR1: soluble form of vascular endothelial growth factor receptor type 1; sVEGFR2: soluble form of vascular endothelial growth factor receptor type 2. Significant differences are marked by bold *p*-values. An underlined *p*-value represents closeness to statistical significance.

**Table 12:** Linear regression models for disease-free survival predictors in breast cancer patients.

		Model 1	Model 2	Model 3	Model 4
Pre-treatment AOPP concentration	Beta *p*-value	0.3695 **0.0018**	0.4041 **0.0007**	0.4912 **<0.0001**	0.3663 **0.0196**
Post-treatment AOPP concentration	Beta *p*-value	0.4737 **<0.0001**	0.4880 **<0.0001**	0.5805 **<0.0001**	0.4490 **0.0015**
Pre-treatment VEGF-A concentration	Beta *p*-value	0.0482 0.7090	0.0934 0.4809	0.0990 0.4640	0.1416 0.2820
Post-treatment VEGF-A concentration	Beta *p*-value	−0.0002 0.9986	0.0498 0.7076	0.0563 0.6744	0.0422 0.7304
Pre-treatment sVEGFR1 concentration	Beta *p*-value	−0.2009 0.1029	−0.2184 0.0774	−0.2862 **0.0198**	−0.2234 0.0593
Post-treatment sVEGFR1 concentration	Beta *p*-value	−0.1003 0.4106	−0.1328 0.2872	−0.1839 0.1413	−0.1222 0.3127
Pre-treatment sVEGFR2 concentration	Beta *p*-value	0.0267 0.8426	0.0037 0.9783	0.0547 0.6890	0.1032 0.4371
Post-treatment sVEGFR2 concentration	Beta *p*-value	−0.0978 0.4269	−0.1279 0.3059	−0.1177 0.3465	−0.0570 0.6405

Model 1 adjusted for age; Model 2 adjusted for age, BMI, parity, menopausal status; Model 3 adjusted for age, BMI, parity, menopausal status, and smoking status; Model 4 adjusted for age, BMI, parity, menopausal status, smoking status, tumour stage, tumour diameters, histological type, nodal involvement. Abbreviations: AOPP: advanced oxidation protein products; VEGF-A: vascular endothelial growth factor A; sVEGFR1: soluble form of vascular endothelial growth factor receptor type 1; sVEGFR2: soluble form of vascular endothelial growth factor receptor type 2. Significant differences are denoted by bold *p*-values; an underlined *p*-value represents closeness to statistical significance.

## Data Availability

The data presented in this study are available on request from the corresponding author.

## Supplementary Materials

The following supporting information can be downloaded at: https://www.mdpi.com/article/10.3390/cancers16051068/s1, Figure S1: A graph showing four ROC curves for the Ki-67 expression, pre- and post-treatment concentrations of AOPP, and tumour diameter for evaluating the most accurate indicator for disease recurrence with different values of area under the curve (AUC) and *p*-values; Figure S2: A graph showing four ROC curves for the Ki-67 expression, pre- and post-treatment concentrations of AOPP, and tumour diameter for evaluating the most accurate indicator for predicting cancer-related death with different values of area under the curve (AUC) and *p*-values.

## References

[1] Sung H., Ferlay J., Siegel R.L., Laversanne M., Soerjomataram I., Jemal A., Bray F. (2021). Global Cancer Statistics 2020: GLOBOCAN Estimates of Incidence and Mortality Worldwide for 36 Cancers in 185 Countries. CA Cancer J. Clin..

[2] Jafari S.H., Saadatpour Z., Salmaninejad A., Momeni F., Mokhtari M., Nahand J.S., Rahmati M., Mirzaei H., Kianmehr M. (2018). Breast Cancer Diagnosis: Imaging Techniques and Biochemical Markers. J. Cell Physiol..

[3] National Cancer Institute Female Breast Cancer: Cancer Stat Facts. https://seer.cancer.gov/statfacts/html/breast.html.

[4] Wang R., Zhu Y., Liu X., Liao X., He J., Niu L. (2019). The Clinicopathological Features and Survival Outcomes of Patients with Different Metastatic Sites in Stage IV Breast Cancer. BMC Cancer.

[5] Madu C.O., Wang S., Madu C.O., Lu Y. (2020). Angiogenesis in Breast Cancer Progression, Diagnosis, and Treatment. J. Cancer.

[6] Ayoub N.M., Jaradat S.K., Al-Shami K.M., Alkhalifa A.E. (2022). Targeting Angiogenesis in Breast Cancer: Current Evidence and Future Perspectives of Novel Anti-Angiogenic Approaches. Front. Pharmacol..

[7] Mdkhana B., Goel S., Saleh M.A., Siddiqui R., Khan N.A., Elmoselhi A.B. (2022). Role of Oxidative Stress in Angiogenesis and the Therapeutic Potential of Antioxidants in Breast Cancer. Eur. Rev. Med. Pharmacol. Sci..

[8] Lugano R., Ramachandran M., Dimberg A. (2020). Tumor Angiogenesis: Causes, Consequences, Challenges and Opportunities. Cell Mol. Life Sci..

[9] Yang F., Jin C., Jiang Y.J., Li J., Di Y., Fu D.L. (2011). Potential Role of Soluble VEGFR-1 in Antiangiogenesis Therapy for Cancer. Expert Rev. Anticancer. Ther..

[10] Bando H., Weich H.A., Brokelmann M., Horiguchi S., Funata N., Ogawa T., Toi M. (2005). Association between Intratumoral Free and Total VEGF, Soluble VEGFR-1, VEGFR-2 and Prognosis in Breast Cancer. Br. J. Cancer.

[11] Cao J., Yang R., Smith T.E., Evans S., McCollum G.W., Pomerantz S.C., Petley T., Harris I.R., Penn J.S. (2019). Human Umbilical Tissue-Derived Cells Secrete Soluble VEGFR1 and Inhibit Choroidal Neovascularization. Mol. Ther. Methods Clin. Dev..

[12] Thielemann A., Baszczuk A., Kopczyński Z., Kopczyński P., Grodecka-Gazdecka S. (2013). Clinical Usefulness of Assessing VEGF and Soluble Receptors SVEGFR-1 and SVEGFR-2 in Women with Breast Cancer. Ann. Agric. Environ. Med..

[13] Gurer-Orhan H., Ince E., Konyar D., Saso L., Suzen S. (2017). The Role of Oxidative Stress Modulators in Breast Cancer. Curr. Med. Chem..

[14] Schetter A.J., Heegaard N.H.H., Harris C.C. (2009). Inflammation and Cancer: Interweaving MicroRNA, Free Radical, Cytokine and P53 Pathways. Carcinogenesis.

[15] Sawicka E., Kratz E.M., Szymańska B., Guzik A., Wesołowski A., Kowal P., Pawlik-Sobecka L., Piwowar A. (2020). Preliminary Study on Selected Markers of Oxidative Stress, Inflammation and Angiogenesis in Patients with Bladder Cancer. Pathol. Oncol. Res..

[16] Komosinska-Vassev K., Olczyk P., Winsz-Szczotka K., Kuznik-Trocha K., Klimek K., Olczyk K. (2012). Age- and Gender-Related Alteration in Plasma Advanced Oxidation Protein Products (AOPP) and Glycosaminoglycan (GAG) Concentrations in Physiological Ageing. Clin. Chem. Lab. Med..

[17] Bagyura Z., Takács A., Kiss L., Dósa E., Vadas R., Nguyen T.D., Dinya E., Soós P., Szelid Z., Láng O. (2022). Level of Advanced Oxidation Protein Products Is Associated with Subclinical Atherosclerosis. BMC Cardiovasc. Disord..

[18] Baskol G., Gumus K., Oner A., Arda H., Karakucuk S. (2018). The Role of Advanced Oxidation Protein Products and Total Thiols in Diabetic Retinopathy. Eur. J. Ophthalmol..

[19] Huang Q.T., Wang S.S., Zhang M., Huang L.P., Tian J.W., Yu Y.H., Wang Z.J., Zhong M. (2013). Advanced Oxidation Protein Products Enhances Soluble Fms-like Tyrosine Kinase 1 Expression in Trophoblasts: A Possible Link between Oxidative Stress and Preeclampsia. Placenta.

[20] Wybranowski T., Napiórkowska M., Bosek M., Pyskir J., Ziomkowska B., Cyrankiewicz M., Pyskir M., Pilaczyńska-Cemel M., Rogańska M., Kruszewski S. (2022). Study of Albumin Oxidation in COVID-19 Pneumonia Patients: Possible Mechanisms and Consequences. Int. J. Mol. Sci..

[21] Zhou C., Zhang Y., Chen J., Mei C., Xiong F., Shi W., Zhou W., Liu X., Sun S., Tian J. (2021). Association between Serum Advanced Oxidation Protein Products and Mortality Risk in Maintenance Hemodialysis Patients. J. Transl. Med..

[22] Liu J., Wen S., Lin Y., Yang X., Liu Z., Quan S., Song Y. (2020). Advanced Oxidation Protein Products Change Biological Behaviors of Rat Endometrial Epithelial Cells by Activating ERK/P38 Signaling Pathways. Biol. Open.

[23] Xian L.-W., Li T.-P., Wei Y.-E., Wu S.-P., Ma L. (2011). Relation of Advanced Oxidation Protein Products with VEGF and TGF-Β1 in Colon Cancer Cells Exposed to Intermittent Hypoxia. Nan Fang Yi Ke Da Xue Xue Bao.

[24] Zhou L.L., Cao W., Xie C., Tian J., Zhou Z., Zhou Q., Zhu P., Li A., Liu Y., Miyata T. (2012). The Receptor of Advanced Glycation End Products Plays a Central Role in Advanced Oxidation Protein Products-Induced Podocyte Apoptosis. Kidney Int..

[25] Lou A., Wang L., Lai W., Zhu D., Wu W., Wang Z., Cai Z., Yang M., Yang M. (2021). Advanced Oxidation Protein Products Induce Inflammatory Responses and Invasive Behaviour in Fibroblast-like Synoviocytes via the RAGE-NF-ΚB Pathway. Bone Joint Res..

[26] Guo Z.J., Niu H.X., Hou F.F., Zhang L., Fu N., Nagai R., Lu X., Chen B.H., Shan Y.X., Tian J.W. (2008). Advanced Oxidation Protein Products Activate Vascular Endothelial Cells via a RAGE-Mediated Signaling Pathway. Antioxid. Redox Signal.

[27] Kim Y.-W., Byzova T.V. (2014). Oxidative Stress in Angiogenesis and Vascular Disease. Blood.

[28] Huang Q., Ji M., Li F., Li Y., Zhou X., Hsueh C., Zhou L. (2023). Diagnostic and Prognostic Value of Plasma Cell-Free DNA Com- bined with VEGF-C in Laryngeal Squamous Cell Carcinoma. Mol. Cell Probes.

[29] Dao J., Conway P.J., Subramani B., Meyyappan D., Russell S., Mahadevan D. (2023). Using CfDNA and CtDNA as Oncologic Markers: A Path to Clinical Validation. Int. J. Mol. Sci..

[30] Cheng F., Su L., Qian C. (2016). Circulating Tumor DNA: A Promising Biomarker in the Liquid Biopsy of Cancer. Oncotarget.

[31] Thierry A.R., El Messaoudi S., Gahan P.B., Anker P., Stroun M. (2016). Origins, Structures, and Functions of Circulating DNA in Oncology. Cancer Metastasis Rev..

[32] Tumburu L., Ghosh-Choudhary S., Seifuddin F.T., Barbu E.A., Yang S., Ahmad M.M., Wilkins L.H.W., Tunc I., Sivakumar I., Nichols J.S. (2021). Circulating Mitochondrial DNA Is a Proinflammatory DAMP in Sickle Cell Disease. Blood.

[33] Dawson S.-J., Tsui D.W.Y., Murtaza M., Biggs H., Rueda O.M., Chin S.-F., Dunning M.J., Gale D., Forshew T., Mahler-Araujo B. (2013). Analysis of Circulating Tumor DNA to Monitor Metastatic Breast Cancer. N. Engl. J. Med..

[34] Qin C., Gu J., Liu R., Xu F., Qian H., He Q., Meng W. (2017). Release of Mitochondrial DNA Correlates with Peak Inflammatory Cytokines in Patients with Acute Myocardial Infarction. Anatol. J. Cardiol..

[35] Alekseeva L., Mironova N. (2021). Role of Cell-Free DNA and Deoxyribonucleases in Tumor Progression. Int. J. Mol. Sci..

[36] Korabecna M., Zinkova A., Brynychova I., Chylikova B., Prikryl P., Sedova L., Neuzil P., Seda O. (2020). Cell-Free DNA in Plasma as an Essential Immune System Regulator. Sci. Rep..

[37] Krychtiuk K.A., Wurm R., Ruhittel S., Lenz M., Huber K., Wojta J., Heinz G., Hülsmann M., Speidl W.S. (2020). Release of Mitochondrial DNA Is Associated with Mortality in Severe Acute Heart Failure. Eur. Heart J. Acute Cardiovasc. Care.

[38] Gaál Kovalčíková A., Janovičová L., Hodosy J., Bábíčková J., Vavrincová-Yaghi D., Vavrinec P., Boor P., Podracká L., Šebeková K., Celec P. (2022). Extracellular DNA Concentrations in Various Aetiologies of Acute Kidney Injury. Sci. Rep..

[39] Barbalata T., Scarlatescu A.I., Sanda G.M., Toma L., Stancu C.S., Dorobantu M., Micheu M.M., Sima A.V., Niculescu L.S. (2022). Mitochondrial DNA Together with MiR-142-3p in Plasma Can Predict Unfavorable Outcomes in Patients after Acute Myocardial Infarction. Int. J. Mol. Sci..

[40] Wang L., Zhang Q., Yuan K., Yuan J. (2021). MtDNA in the Pathogenesis of Cardiovascular Diseases. Dis. Markers.

[41] Demkow U. (2023). Molecular Mechanisms of Neutrophil Extracellular Trap (NETs) Degradation. Int. J. Mol. Sci..

[42] Papayannopoulos V. (2017). Neutrophil Extracellular Traps in Immunity and Disease. Nat. Rev. Immunol..

[43] Arnhold J. (2020). The Dual Role of Myeloperoxidase in Immune Response. Int. J. Mol. Sci..

[44] Rizo-Téllez S.A., Sekheri M., Filep J.G. (2022). Myeloperoxidase: Regulation of Neutrophil Function and Target for Therapy. Antioxidants.

[45] Descamps-Latscha B., Witko-Sarsat V. (2001). Importance of Oxidatively Modified Proteins in Chronic Renal Failure. Kidney Int. Suppl..

[46] Capeillère-Blandin C., Gausson V., Descamps-Latscha B., Witko-Sarsat V. (2004). Biochemical and Spectrophotometric Significance of Advanced Oxidized Protein Products. Biochim. Biophys. Acta Mol. Basis Dis..

[47] Yue Q., Song Y., Liu Z., Zhang L., Yang L., Li J. (2022). Receptor for Advanced Glycation End Products (RAGE): A Pivotal Hub in Immune Diseases. Molecules.

[48] Fink K., Boratyński J. (2012). The Role of Metalloproteinases in Modification of Extracellular Matrix in Invasive Tumor Growth, Metastasis and Angiogenesis. Adv. Hyg. Exp. Med..

[49] Witko-Sarsat V., Friedlander M., Capeillere-Blandin C., Nguyen-Khoa T., Nguyen A.T., Zingraff J., Jungers P., Descamps-Latscha B. (1996). Advanced Oxidation Protein Products as a Novel Marker of Oxidative Stress in Uremia. Kidney Int..

[50] Hanasand M., Omdal R., Norheim K.B., Gøransson L.G., Brede C., Jonsson G. (2012). Improved Detection of Advanced Oxidation Protein Products in Plasma. Clin. Chim. Acta.

[51] Zarychta E., Rhone P., Bielawski K., Rosc D., Szot K., Zdunska M., Ruszkowska-Ciastek B. (2018). Elevated Plasma Levels of Tissue Factor as a Valuable Diagnostic Biomarker with Relevant Efficacy for Prediction of Breast Cancer Morbidity. J. Physiol. Pharmacol..

[52] American Cancer Society About Breast Cancer. https://www.cancer.org/content/dam/CRC/PDF/Public/8577.00.pdf.

[53] Abd El-Khalik S.R., Hafez Y.M., Elkholy R.A. (2020). The Role of Circulating Soluble Fms-like Tyrosine Kinase-1 in Patients with Diabetic Foot Ulcer: A Possible Mechanism of Pathogenesis via a Novel Link between Oxidative Stress, Inflammation and Angiogenesis. Microvasc. Res..

[54] Kundaktepe B.P., Sozer V., Durmus S., Kocael P.C., Kundaktepe F.O., Papila C., Gelisgen R., Uzun H. (2021). The Evaluation of Oxidative Stress Parameters in Breast and Colon Cancer. Medicine.

[55] Kilic N., Yavuz Taslipinar M., Guney Y., Tekin E., Onuk E. (2014). An Investigation into the Serum Thioredoxin, Superoxide Dismutase, Malondialdehyde, and Advanced Oxidation Protein Products in Patients with Breast Cancer. Ann. Surg. Oncol..

[56] Salehi S.S., Mirmiranpour H., Rabizadeh S., Esteghamati A., Tomasello G., Alibakhshi A., Najafi N., Rajab A., Nakhjavani M. (2021). Improvement in Redox Homeostasis after Cytoreductive Surgery in Colorectal Adenocarcinoma. Oxid. Med. Cell. Longev..

[57] Koike A., Robles B.E.F., da Silva Bonacini A.G., de Alcantara C.C., Reiche E.M.V., Dichi I., Maes M., Cecchini R., Simão A.N.C. (2020). Thiol Groups as a Biomarker for the Diagnosis and Prognosis of Prostate Cancer. Sci. Rep..

[58] Otsmane A., Kacimi G., Adane S., Cherbal F., Aouichat Bouguerra S. (2018). Clinico-Epidemiological Profile and Redox Imbalance of Lung Cancer Patients in Algeria. J. Med. Life.

[59] Kosova F., Çetin B., Akinci M., Aslan S., Ari Z., Sepici A., Altan N., Çetin A. (2007). Advanced Oxidation Protein Products, Ferrous Oxidation in Xylenol Orange, and Malondialdehyde Levels in Thyroid Cancer. Ann. Surg. Oncol..

[60] Brogowska K.K., Zajkowska M., Mroczko B. (2023). Vascular Endothelial Growth Factor Ligands and Receptors in Breast Cancer. J. Clin. Med..

[61] Huang Y.J., Nan G.X. (2019). Oxidative Stress-Induced Angiogenesis. J. Clin. Neurosci..

[62] Suvakov S., Jerotic D., Damjanovic T., Milic N., Pekmezovic T., Djukic T., Jelic-Ivanovic Z., Savic Radojevic A., Pljesa-Ercegovac M., Matic M. (2019). Markers of Oxidative Stress and Endothelial Dysfunction Predict Haemodialysis Patients Survival. Am. J. Nephrol..

[63] Chiang F.F., Chao T.H., Huang S.C., Cheng C.H., Tseng Y.Y., Huang Y.C. (2022). Cysteine Regulates Oxidative Stress and Glutathione-Related Antioxidative Capacity before and after Colorectal Tumor Resection. Int. J. Mol. Sci..

[64] Ek R.O., Yildiz Y., Cecen S., Yenisey C., Kavak T. (2008). Effects of Tamoxifen on Myocardial Ischemia-Reperfusion Injury Model in Ovariectomized Rats. Mol. Cell. Biochem..

[65] Silva F.B., Romero W.G., Carvalho A.L.R.D.A., Souza G.A.A., Claudio E.R.G., Abreu G.R. (2017). Effects of Treatment with Chemotherapy and/or Tamoxifen on the Biomarkers of Cardiac Injury and Oxidative Stress in Women with Breast Cancer. Medicine.

[66] Baranova A., Krasnoselskyi M., Starikov V., Kartashov S., Zhulkevych I., Vlasenko V., Oleshko K., Bilodid O., Sadchikova M., Vinnyk Y. (2022). Triple-Negative Breast Cancer: Current Treatment Strategies and Factors of Negative Prognosis. J. Med. Life.

[67] Li Y., Ma L. (2020). Efficacy of Chemotherapy for Lymph Node-Positive Luminal A Subtype Breast Cancer Patients: An Updated Meta-Analysis. World J. Surg. Oncol..

[68] Hennigs A., Riedel F., Gondos A., Sinn P., Schirmacher P., Marmé F., Jäger D., Kauczor H.U., Stieber A., Lindel K. (2016). Prognosis of Breast Cancer Molecular Subtypes in Routine Clinical Care: A Large Prospective Cohort Study. BMC Cancer.

[69] Abdou Y., Gupta M., Asaoka M., Attwood K., Mateusz O., Gandhi S., Takabe K. (2022). Left Sided Breast Cancer Is Associated with Aggressive Biology and Worse Outcomes than Right Sided Breast Cancer. Sci. Rep..

[70] Nelson D.R., Brown J., Morikawa A., Method M. (2022). Breast Cancer-Specific Mortality in Early Breast Cancer as Defined by High-Risk Clinical and Pathologic Characteristics. PLoS ONE.

[71] Zhou F.L., Zhang W.G., Wei Y.C., Meng S., Bai G.G., Wang B.Y., Yang H.Y., Tian W., Meng X., Zhang H. (2010). Involvement of Oxidative Stress in the Relapse of Acute Myeloid Leukemia. J. Biol. Chem..

[72] Delrieu L., Touillaud M., Pérol O., Morelle M., Martin A., Friedenreich C.M., Mury P., Dufresne A., Bachelot T., Heudel P.E. (2021). Impact of Physical Activity on Oxidative Stress Markers in Patients with Metastatic Breast Cancer. Oxid. Med. Cell. Longev..

[73] Ben Mahmoud L., Mdhaffar M., Ghozzi H., Ammar M., Hakim A., Atheymen R., Sahnoun Z., Elloumi M., Zeghal K. (2017). Oxidative Stress in Tunisian Patients with Acute Lymphoblastic Leukemia and Its Involvement in Leukemic Relapse. J. Pediatr. Hematol. Oncol..

[74] Kaneda H., Taguchi J., Ogasawara K., Aizawa T., Ohno M. (2002). Increased Level of Advanced Oxidation Protein Products in Patients with Coronary Artery Disease. Atherosclerosis.

[75] Cai J., Han T., Nie C., Jia X., Liu Y., Zhu Z., Gao Y. (2016). Biomarkers of Oxidation Stress, Inflammation, Necrosis and Apoptosis Are Associated with Hepatitis B-Related Acute-on-Chronic Liver Failure. Clin. Res. Hepatol. Gastroenterol..

[76] Elkabany Z.A., El-Farrash R.A., Shinkar D.M., Ismail E.A., Nada A.S., Farag A.S., Elsayed M.A., Salama D.H., Macken E.L., Gaballah S.A. (2020). Oxidative Stress Markers in Neonatal Respiratory Distress Syndrome: Advanced Oxidation Protein Products and 8-Hydroxy-2-Deoxyguanosine in Relation to Disease Severity. Pediatr. Res..

