# Supplementary material for: A Preliminary Evaluation of Advanced Oxidation Protein Products (AOPPs) as a Potential Approach to Evaluating Prognosis in Early-Stage Breast Cancer Patients and Its Implication in Tumour Angiogenesis: A 7-Year Single-Centre Study
Source: Cancers (Basel). 2024 Mar 6;16(5):1068. doi: 10.3390/cancers16051068 (PMC10930735; doi:10.3390/cancers16051068)
Supplement: Supplementary file 1 [file cancers-16-01068-s001.zip › cancers-2862867-supplementary.pdf]

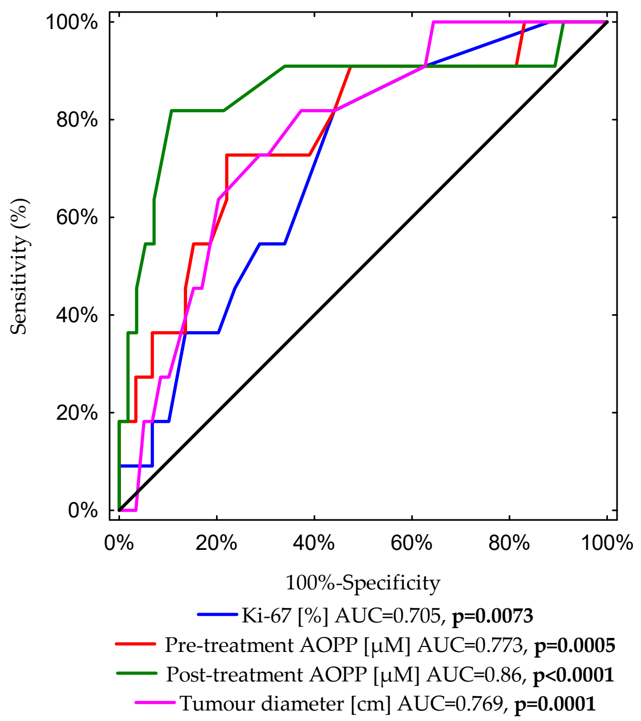

Figure S1 A graph showing four ROC curves for the Ki-67 expression, pre-and post-treatment concentrations of AOPP, and tumour diameter for evaluating the most accurate indicator for disease recurrence with different values of area under the curve (AUC) and *p*-values.

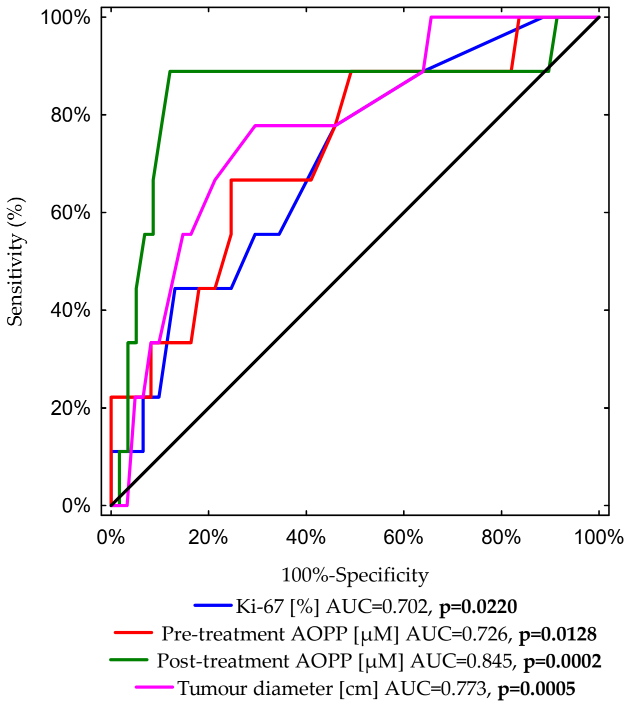

Figure S2 A graph showing four ROC curves for the Ki-67 expression, pre-and post-treatment concentrations of AOPP, and tumour diameter for evaluating the most accurate indicator for predicting cancer-related death with different values of area under the curve (AUC) and *p*-values.
